# Comprehensive Review of Synthesis, Optical Properties and Applications of Heteroarylphosphonates and Their Derivatives

**DOI:** 10.3390/molecules29153691

**Published:** 2024-08-04

**Authors:** Krzysztof Owsianik, Adrian Romaniuk, Marika Turek, Piotr Bałczewski

**Affiliations:** 1 Division of Organic Chemistry, Center of Molecular and Macromolecular Studies, Polish Academy of Sciences, Sienkiewicza 112, 90-363 Łódź, Poland; adrian.romaniuk@cbmm.lodz.pl; 2 Institute of Chemistry, Faculty of Science and Technology, Jan Długosz University in Częstochowa, Armii Krajowej 13/15, 42-200 Częstochowa, Poland; m.turek@ujd.edu.pl

**Keywords:** heteroarylphosphonate, semiconductor, OLED, UV-Vis absorption, fluorescence, ruthenium complex, lanthanide complex, pyridylphosphonate

## Abstract

This review focuses on optical properties of compounds in which at least one phosphonate group is directly attached to a heteroaromatic ring. Additionally, the synthesis and other applications of these compounds are addressed in this work. The influence of the phosphonate substituent on the properties of the described compounds is discussed and compared with other non-phosphorus substituents, with particular attention given to photophysical properties, such as UV-Vis absorption and emission, fluorescence quantum yield and fluorescence lifetime. Considering the presence of heteroatom, the collected material was divided into two parts, and a review of the literature of the last thirty years on heteroaryl phosphonates containing sulfur and nitrogen atoms in the aromatic ring was conducted.

## 1. Introduction

Heteroaryl- and arylphosphonates are useful compounds with a wide range of applications in various fields of chemistry and materials science, especially due to their valuable optical properties, so a look at them from this angle is the subject of this review [1,2]. These compounds are particularly notable for their use as photosensitizers in water oxidation processes, specifically as ligands in ruthenium(II) polypyridyl complexes [3]. The properties of these complexes can be finely tuned by substituting bipyridine ligands, resulting in enhanced performance and functionality. In the field of semiconductor technology, heteroarylphosphonates are used to produce nanocrystalline semiconductors. Moreover, ruthenium complexes incorporating heteroarylphosphonates serve as reliable sources of photoexcited electrons in molecular devices, finding applications in organic solar cells, fluorescent cation sensors, and other energy-transfer systems. The phosphonium groups also function as anchoring groups in nanocrystalline semiconductors without adversely affecting the optical properties of the light-emitting dyes. In analytical biochemistry, lanthanide complexes featuring 2-pyridylphosphonic acid as ligands are gaining importance, particularly in nuclear magnetic resonance imaging (MRI) [4]. The formation of polynuclear species in solution can be exploited to create complex heteropolynuclear structures, enhancing the utility of these ligands. Heteroarylphosphonates also shine in the development of lanthanide-containing luminescent probes for analytical sensors, medical analyses and living cell or tissue imaging [5,6]. Their solubility in water, high quantum yield and suitability for multiphoton excitation make them particularly attractive. Additionally, the strong co-ordination ability of phosphonic acids enhances the thermodynamic stability of these complexes. In the field of optoelectronics, heteroarylphosphonates serve as deep-blue, fluorescent emitters for efficient single-layer organic light-emitting diodes (OLEDs), polymer light-emitting diodes (PLEDs) and perovskite quantum dot light-emitting diodes (QD-LEDs) [7,8]. They also exhibit notable properties in proton-coupled electron transfer (PCET), which is a useful mechanism for reducing the redox potentials of high-valent transition metal complexes. The diverse substituents on heteroarylphosphonates make them valuable electrochemical and photochemical modifiers, enhancing the performance of photosensitized solar cells where Ru-bipyridine dyes are anchored to TiO_2_ surfaces via carboxylic and phosphonic acid groups [9]. Additionally, these compounds show promise as luminescent materials for sensing metal ions [10]. This broad spectrum of applications underscores the significance of heteroarylphosphonates in advancing both fundamental research and technological innovation, and motivates the discovery of new synthetic methods for these compounds. Therefore, this was the reason for the interest in this group of compounds. However, there are no reports in the literature on this topic before 1994, hence this comprehensive review covers the period of 1994–2024.

## 2. Optical Properties, Synthesis and Applications of Heteroarylphosphonates and Their Derivatives

### 2.1. Heteroarylphosphonates with Heteroaryl Sulfur Atom

Oligothiophenes are a promising class of fluorescent molecules that have potential applications in photovoltaics and fluorescence sensing [11,12]. They are employed as conducting molecules because of their favorable optical and electronic properties, as well as their stability. Electronic properties of oligothiophenes are tuneable based on the number of conjugated subunits and types of substituents on the subunits.

**Scheme 1:**
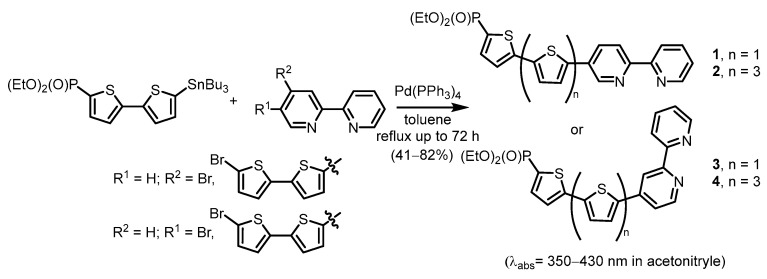
Synthesis of bithiophenes and tetrathiophenes **1**–**4**.

**Figure 1:**
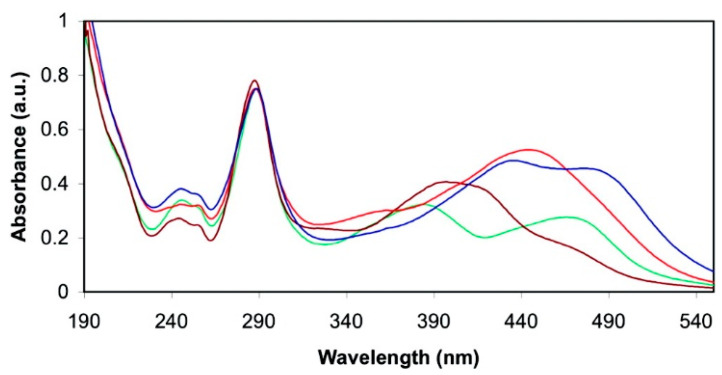
Absorbance spectra of the four Ru-oligothiophenes (Ru-**1**, brown; Ru-**2**, red; Ru-**3**, green; Ru-**4**, blue; 0.01 mM in acetonitrile) (Reprinted with permission from [13], Copyright 2007 American Chemical Society).

Thiophene-thiophene cross-coupling reactions have been used by Bair et al. for the synthesis of unsymmetrical bithiophenes and tetrathiophenes **1**–**4** containing bipyridine and phosphonic group (Scheme 1) [13]. The ligands themselves revealed absorbance at 240 nm from thiophene, 275–290 nm from bipyridine and 350–430 nm assigned to a π→π* transition due to the entire thienylbipyridine unit. However, when attached to ruthenium, both their fluorescence and that of ruthenium were quenched. An additional effect of the co-ordination of ruthenium to compounds **1**–**4** via nitrogen atoms was the appearance of a new absorption band at 470 nm (Figure 1).

**Scheme 2:**
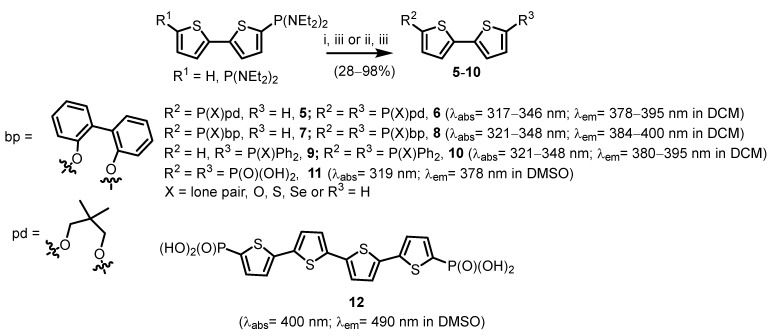
Synthesis of chalcogenophosphonato-substituted bithiophenes. Reagents and conditions: (i) 2,2′-biphenol or 2,2-dimethyl-1,3-propanediol, toluene, reflux; (ii) (a) BuLi, −78 °C, THF; (b) Ph_2_PCl, 0 °C—rt, 2 h; (iii) (X = O) H_2_O_2_·urea complex or N(O)Me_3_; (X = S) S_8_; (X = Se) Se powder, DCM, rt.

The P(III) and P(IV)(O, S, Se)-substituted bithiophenes **5**–**8** were synthesized in low-to-good yields (28–92%) (Scheme 2). The compared fluorescence quantum yields formed a series: **7** (27%, X = S) > **10** (21%, X = S) > **9**, **10** (15–17%, X = O) [14], and they were significantly improved compared to 2,2′-bithiophene. Each compound showed a broad absorption band ranging from 300 to 400 nm and a less intense band at higher energy (250–270 nm), assigned to the π→π* transition of the bithiophene ring system. The difference in the energy of the linear absorption band upon changing the phosphorus substituents was less than or equal to 9 nm, implying that there was little phosphorus resonance contribution to aromatic systems. There are no simple rules relating the emission properties to the chemical structures for this class of molecules. This is in contrast to the structure–property relationships for the absorption spectra, which showed distinct additive trends with structure changes. The compounds showed optical transparencies above 420 nm for **5**, **6** or 430 nm for **7**, **8**, making them suitable candidates for optical power limiting (OPL) measurements at wavelengths across the violet-blue spectral region [15].

Both phosphonic acids **11** and **12** were selected for studies on the dynamic response in layered assemblies because the emission band of **11** (350–550 nm) coincided with the absorption band of **12** (300–450 nm) (Scheme 2). Such overlapping spectra of two chromophores and the possibility of photoisomerization through ring rotation are useful in excitation transport studies [16,17].

**Scheme 3:**
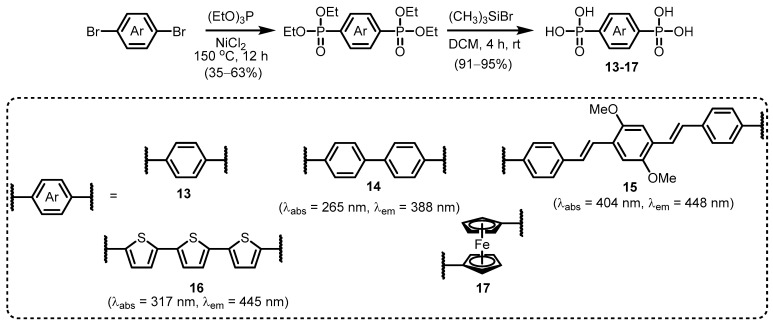
Synthesis of bis(aryl- and heteroaryl)phosphonic acids **13**–**17**.

Chan, Luh and co-workers have described the construction of the first organic–inorganic hybrid materials (OIHM) from phosphonic acids and aluminum lactate [18]. It is expected that these materials should act similarly to silicon-based materials and could be used in photovoltaic applications. The authors synthesized a series of phosphonic acids by coupling triethylphosphite with corresponding aryl bromides to obtain phosphonates. The phosphonates were then hydrolyzed with trimethylsilyl bromide, yielding phosphonic acids **13**–**17** (Scheme 3).

The absorption and emission peaks were measured for the compounds **14**–**16**, and their wavelengths increased in the series **14**, **16** and **15**, which could be rationalized by the raising π-conjugation. The NMR studies have shown that the OIHM exist as aluminum-phosphorus salts, i.e., [Al-OP(O)Ar-OH] or [Al-OP(O)ArO-Al]. Further photochemical studies have shown that aluminum films made of **14** and **15** exhibit fluorescence resonance energy transfer (FRET) when the molar fraction of **14** exceeded 0.9. The complex of four equivalents of aluminum lactate and one equivalent of **15** were measured as having the highest fluorescence lifetime.

**Figure 2:**
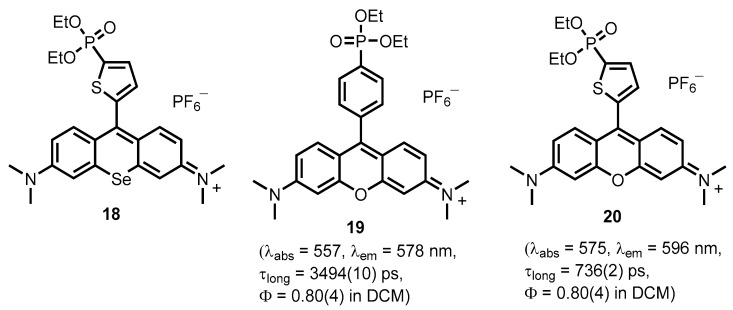
Chemical structures of chalcogenorhodamine dyes **18**–**20**.

**Scheme 4:**
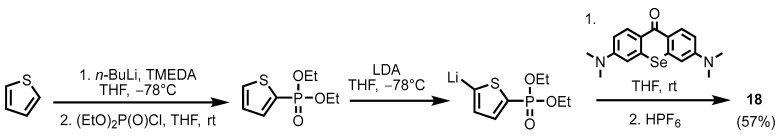
The synthesis of compound **18**.

Detty, Eisenberg, McCamant et al. synthesized a set of chalcogenorhodamine dyes **18**–**20** according to the previously reported procedures [19], in which the ring heteroatoms were O or Se (Figure 2) [20]. The goal of the study was to deepen the understanding of the photophysical properties of chalcogenorhodamine dyes. The procedure is illustrated by the synthesis of compound **18** (Scheme 4).

Compounds **19**–**20** exhibited red-shift when compared to the unsubstituted compounds. All the compounds were relatively stable, with moderate lifetimes. The authors rationalized their photochemical findings by assigning them to an excited state with charge separation.

### 2.2. Heteroarylphosphonates with Heteroaryl Nitrogen

Photophysical properties and diverse applications (optoelectronics, metal ion detection, imaging tools, fluorescence probes, photosensitisers, catalysis, etc.) of compounds with heteroaryl nitrogen are widely described in the literature [21,22,23,24,25,26]. The presence of donor atoms in the molecule, such as nitrogen in the heteroaromatic system and oxygen atoms in the phosphonate group, enables the formation of co-ordination complexes, therefore, the vast majority of compounds presented in this part are ligands and their complexes, particularly with d^10^ metal ions, which generated significant interest due to their photoluminescent properties. Presented ligands are also highly effective at chelating lanthanide cations due to their strong electrostatic interactions and near-neutral second pKa values. Compared to carboxylates, phosphonates are bulkier and provide greater steric hindrance, which is beneficial for shielding luminescent Ln cations from water molecules that cause luminescence loss through nonradiative quenching. Heteroarylphosphonates were also used as electron injectors in PLED devices or chromophore sensitizers.

#### 2.2.1. Pyridylphosphonic Acids and Derivatives

**Figure 3:**
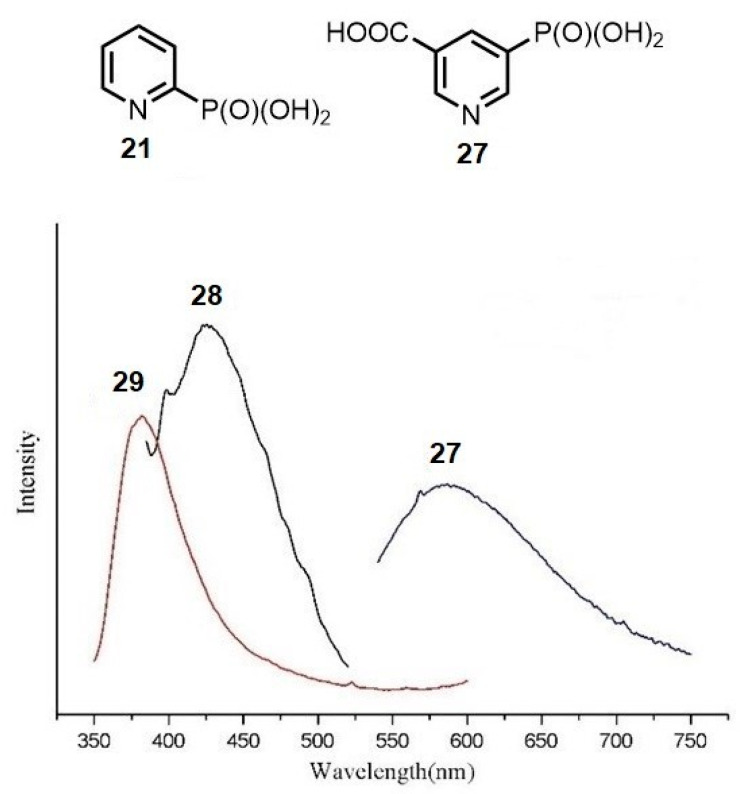
Emission spectra of complex Cd_2_[OOCC_5_H_3_NPO_3_H]_2_·H_2_O (**28**) (*λ*_ex_ = 323 nm), Zn[OOCC_5_H_4_NPO_3_]·H_2_O (**29**) (λ_ex_ = 365 nm) and free ligand **27** (λ_ex_ = 521 nm) (reprinted with permission from [28], Copyright Taylor and Francis Ltd., http://www.tandfonline.com).

Metal-organic polymers constructed from metallic clusters are currently of interest due to their intriguing molecular structures and crystal packing patterns, as well as their potential for custom-designed functionalities. Besides being durable and thermally stable, some exhibit photoluminescent properties, making d^10^ metal polynuclear clusters a focus in the quest for new materials. Five polymers based on metal complexes of **93** were prepared and their room-temperature luminescence was probed (Figure 3) [27]. The reaction of (2-pyridyl)phosphonic acid (2pypo) (**21**) with ZnX_2_ (X = Br, Cl), CdCl_2_, Hg(NO_3_)_2_, or Ag(SO_3_CF_3_) afforded corresponding polymers [Zn(X)(2pypo)]*_n_* {X = Cl = (**22**), X = Br = (**23**)}, [Cd(μ-Cl)_2_(2pypo)]*_n_* (**24**), [Hg(2pypo)]*_n_*·H_2_O (**25**) and [Ag(SO_3_CF_3_)(2pypo)]*_n_* (**26**) a moderate yield from 22% to 81%. Emission of the free ligand was recorded at excitation wavelengths 250, 275, 300, 350 and 375 nm, and emission wavelengths were recorded at 306 and 331 nm. Polymers **22**–**26** showed no luminescence at ambient temperature, whereas polymer **26**, when dissolved in acetonitrile, exhibited a weak emission at 313 nm. It was thought that room-temperature luminescence was too weak to be detected and required low-temperature measurements.

Based on 5-phosphononicotinic acid (**27**), two transition metal phosphonates, Cd_2_[OOCC_5_H_3_NPO_3_H]_2_·H_2_O (**28**) and Zn[OOCC_5_H_4_NPO_3_]·H_2_O (**29**), were synthesized. The compound **28** emitted a purple-blue light upon 323 nm excitation. Above 100 nm, blue shifts have been observed for both compounds (Figure 3) [28].

**Scheme 5:**
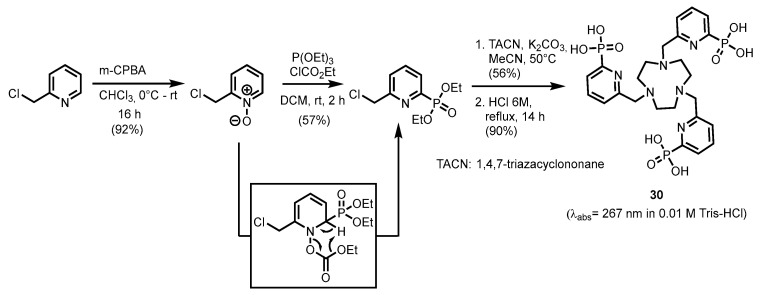
Synthesis of the ligand **30**.

Charbonnière and co-workers have presented complexes with ligands based on aza-crown ethers functionalized with phosphonate groups [29]. They were interested in obtaining a system in which photonic up-conversion, i.e., conversion of low-energy photons to visible ones, could take place. The synthesis of ligand **30** involved a single decarboxylation and P-functionalization step. It was achieved by the oxidation of the pyridine derivative to pyridine *N*-oxide and subsequent treatment with ethyl chloroformate and triethyl phosphite. Then, chloride moieties were substituted with nitrogen atoms from 1,4,7-triazacyclononane followed by hydrolysis of all phosphonate ester groups to form **30** (Scheme 5).

It has been shown that ligand **30** formed stable LnL complexes with Ln = La, Lu, Eu, Tb and Yb. Ligand **30** exhibited an absorption peak at 267 nm (in 0.01 M Tris-HCl), which could be attributed to $\pi \to \pi^{*}$ transitions of the pyridine phosphonate group. It has also been proven that Yb, Tb and Eu complexes showed emission peaks at approximately 1000, 550 and 620 nm, respectively rendering them highly luminescent. The formation of polynuclear complexes [(LnL)_2_Ln_x_] for x = 1, 2, 3 was driven by the strong polarization of the ligand due to the electron-rich phosphonated pendant arms. For example, the deuterated analogue YbL_2_D used in the formation of [(YbL_2_D)_2_Tb_x_] complexes (x = 1–3) afforded characteristic visible UC emission of Tb upon NIR excitation of the Yb at 980 nm.

**Scheme 6:**
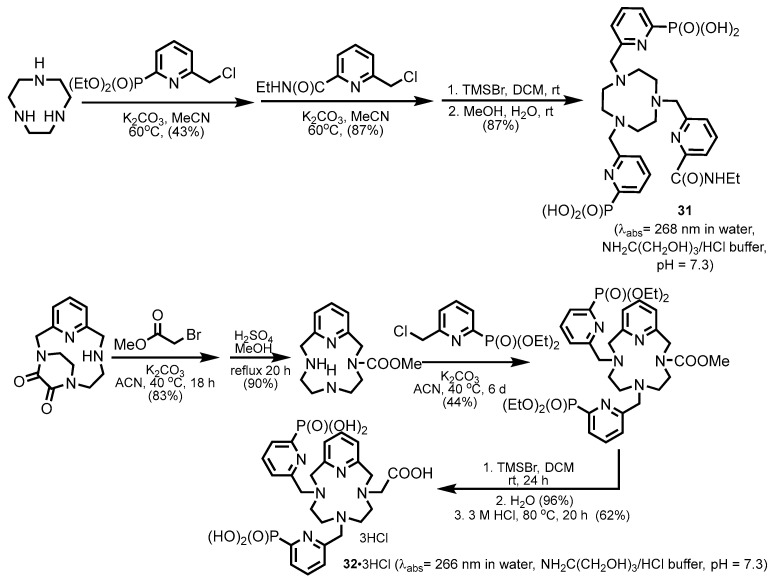
Synthesis of ligands **31** and **32**.

Synthetic methodologies have been developed for the preparation of ligands **31** and **32**, which were chelators based on 1,4,7-triazacyclonane and pyclene (Scheme 6) [30]. Both ligands were obtained in high yields and utilized in a comprehensive photophysical study involving complexation with Eu^3+^, Tb^3+^ and Yb^3+^. The addition of the solution of the Yb^3+^ salt resulted in a bathochromic shift of the maximum from 268 to 271 nm, together with the appearance of a shoulder at ca. 275 nm, characteristic of complexation of the pyridyl phosphonate moieties. Similar results were observed in the case of Eu^3+^ and Tb^3+^. Spectroscopic characterization of the Ln^3+^ complexes of **31** and **32** was done in water and D_2_O, and showed the effective sensitization of the lanthanide metal-centered emission spectra, each exhibiting typical lanthanide emission bands.

**Scheme 7:**
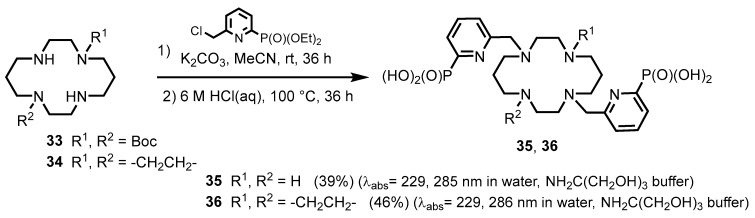
Synthetic protocol for the preparation of ligands **35** and **36**.

The synthesis of the two ligands **35** and **36** from amines **33** and **34,** and then the formation of heteropolymetallic complex Lu-**35,** were presented by Charbonnière et al. (Scheme 7) [31]. This spectroscopically silent lutetium analogue was used to determine the emissive properties of the ligand **35**. From the phosphorescent emission measured at 77 K, the energy level of the triplet state was determined to be 27,930 cm^−1^, and the corresponding lifetime was found to be 462 ± 2 ms. A series of lanthanide complexes was prepared with **35**, and the solution, structural, potentiometric and photophysical properties of these complexes were investigated.

**Scheme 8:**
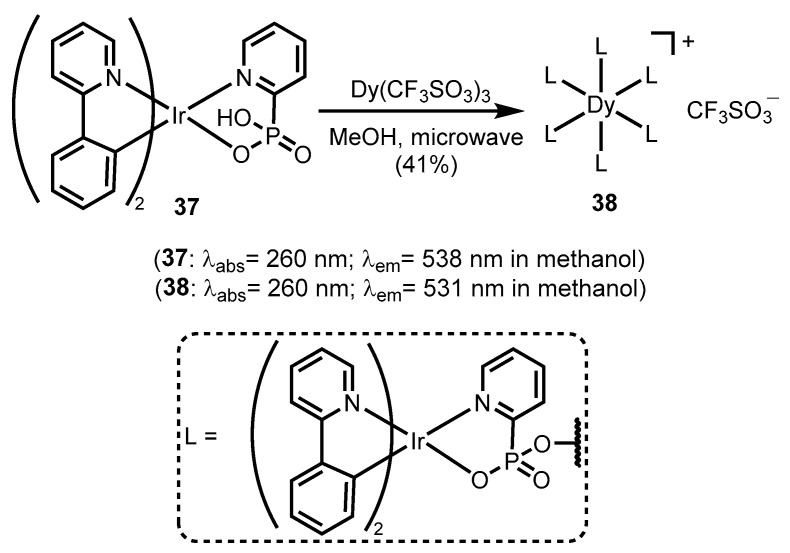
Synthesis of iridium complex **38** from complex **37**.

Zheng et al. published the first example of iridium/dysprosium phosphonate for single-molecule magnets (SMMs) and photoelectronics [32]. Complex **38** showed both field-induced slow magnetization relaxation and photoluminescence, so it could be successfully used in SMMs and photoelectronics (Scheme 8). The synthesis was straightforward and could be accomplished by the treatment of an iridium-phosphonic acid complex with dysprosium(III) trifluoromethanesulfonate, which yielded compound **38**.

The absorption spectra showed that **38** exhibited six times more intensive absorption than **37**. Furthermore, emission spectra suggested that **38** was slightly more blue-shifted than **37**.

**Figure 4:**
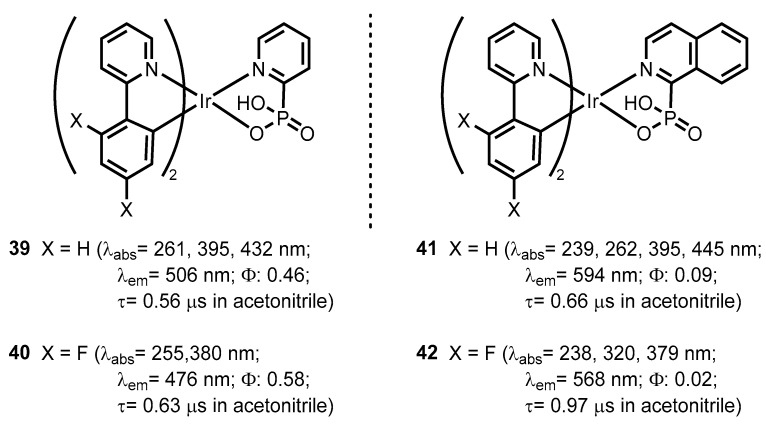
Chemical structures of iridium complexes **39**–**42**.

Zheng, Ma and co-workers synthesized the series of iridium complexes **39**–**42** containing the phosphonic acid moiety for usage in photoelectronics [33]. Moreover, complexes **41** and **42** contained a fluorine atom as well (Figure 4).

The synthesis involved simple complexation of Ir(2-(2-pyridinyl)phenyl)Cl_2_ with a corresponding pyridine derivative, prepared according to the procedure in the literature. In emission spectra studies, complex **39** showed a yellow emission peak at 506 nm, while compound **40** was significantly red-shifted and its peak intensity was about 10 times lower than that of **39**. Similarly for **41** and **42**, the emission peaks were at 476 nm and 568 nm, respectively.

**Figure 5:**
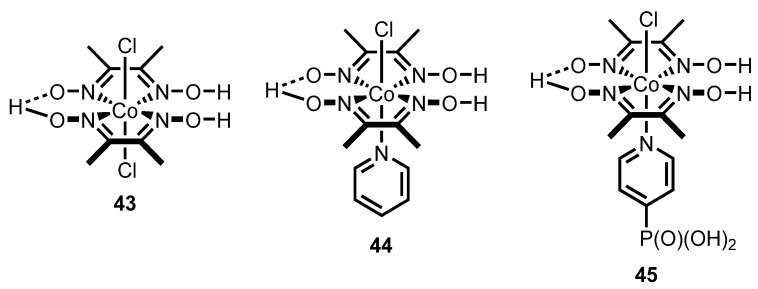
Chemical structures of complexes **43**–**45**.

Hollow-core photonic crystal fibers (HC-PCFs) provide a novel approach for in situ UV-Vis spectroscopy with enhanced detection sensitivity. Euser et al. demonstrated that longer optical path lengths than those afforded by conventional cuvette-based UV-Vis spectroscopy can be used to detect and identify the Co(I) and Co(II) states in hydrogen-evolving cobaloxime catalysts with a Ru(II)-based photosensitizer. Cobalt complexes with various groups in the axial position, such as the chloride group [CoCl_2_(dmgH)(dmgH_2_)] (dmg: dimethylglyoxime) **43**, the pyridyl group [CoCl(dmgH)_2_py] **44** and the pyridyl group with phosphonate substituent **45**, were selected as model compounds for these studies (Figure 5) [34,35].

**Figure 6:**
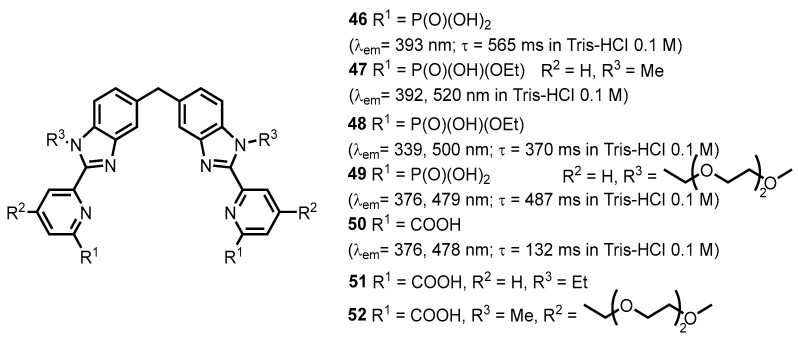
Chemical structures of ligands **46**–**52**.

Luminescent lanthanide helicates, self-assembled from ditopic ligands **46**–**49** bearing phosphonic acid or phosphoester units, were studied by Bünzli et al. (Figure 6) [36]. Photophysical measurements revealed sensitization of the metal-centered luminescence in the europium and terbium complexes, which was modulated by the nature of the ligand. The absorption spectra of the deprotonated ligands **46**–**52** feature two main bands located around 310 nm (32,260 cm^−1^), 217 nm (46,080 cm^−1^) and a shoulder at 240 nm (41,670 cm^−1^), mainly involving orbitals located on the aromatic rings and phosphoryl groups. At 295 K, UV excitation in the π → π* absorption bands results in a broad emission band with a maximum between 25,500 and 29,500 cm^−1^ for the ligands, and between 24,250–28,500 cm^−1^ for the complexes with Lu and Gd.

The **46**–**49** series of ligands acted similarly to the **50**–**52** ligands with regard to the co-ordination of the lanthanide ion (Ln = Eu, Gd, Tb), and the replacement of the carboxylates by a phosphoester substituent on the pyridine ring was a valuable alternative to the grafting of a poly(oxyethylene) arm with respect to solubility. Preliminary results indicated that [Eu_2_(**47**)_3_] did indeed penetrate into live *HeLa* cells and were potential candidates for lanthanide luminescent probes.

**Figure 7:**
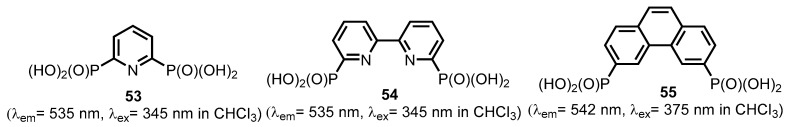
Chemical structures of *N,O*-donor *N*-heterocyclic aromatic diphosphonate ligands **53**–**55**.

Pyridine diphosphonate ligand **53** was used for the stabilization of tetravalent uranium and neptunium in an aqueous medium under aerobic conditions (Figure 7). Raman, UV-Vis, fluorescence and cyclic voltametric studies, along with density functional theory (DFT) calculations, were conducted to obtain structural insights into these complexes in different oxidation states [37].

#### 2.2.2. Bipyridinephosphonic Acids and Derivatives

Phosphonate ligands are widely used for extracting ‘f’ block elements. Three *N*,*O*-donor *N*-heterocyclic aromatic diphosphonate ligands **53**–**55** were tested for Am^3+^ and Eu^3+^ extraction/separation (Figure 7) [38]. Luminescence titration in an aqueous medium showed formation of the anionic complex with Eu^3+^. Liquid–liquid extraction with Aliquat-336 revealed the 1:3 metal–ligand stoichiometry for the acid **53**, the 1:2 metal–ligand stoichiometry for acid **54**, and both 1:2 and 1:3 stoichiometry for the acid **55**. DFT calculations predicted their structures, and electrospray ionization mass spectrometry confirmed the calculations. Non-selectivity towards Am^3+^ and Eu^3+^ was due to unfavorable covalent interactions in the metal–ligand bonds.

**Figure 8:**
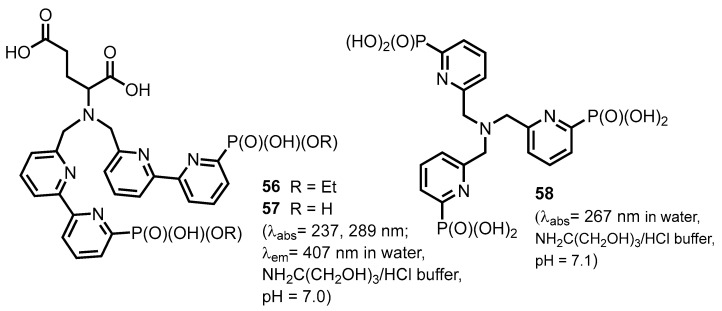
Chemical structures of ligands **56**–**58**.

The synthesis and characterization of three ligands **156**, **57** and **58** with phosphonic groups were described by Charbonnière et al. (Figure 8). As has been shown, in contrast to acid **57**, which acted as a heptadentate ligand, ligand **56** did not form stable complexes with lanthanide cations in aqueous solutions (0.01 M Tris/HCl, pH 7.0). Probably, the negative charge brought by the phosphonate functions largely counterbalanced the positive charge of the lanthanide cations. The UV-visible absorption spectrum of ligand **57** in water showed distinct transitions at 237 and 289 nm, accompanied by a shoulder at 315 nm. Excitation at 320 nm resulted in emission at 407 nm, attributed to ^1^π→π* transitions. Upon complexation with lanthanides, absorption bands shifted, suggesting ligand-to-metal sensitization. However, the resulting metal-centered luminescence displayed low quantum yields compared to reference complexes [39]. Phosphonic acid **58** can form stable complexes with lanthanide cations, which form polynuclear species in the presence of an excess of lanthanides. The UV-Vis absorption spectrum of the free ligand was dominated by the intense π→π* transitions of pyridine units at 267 nm (*ε* = 14,700 M^−1^ cm^−1^) [4,40].

Polypyridyl complexes, whose properties can be easily tuned by substituting bipyridine ligands with phosphonate groups, are used as photosensitizers. Such compounds can play a distinctive role in catalysis and significantly affect the efficiency of water oxidation to produce hydrogen using sunlight. They should provide visible light absorption, a long-lived excited state to improve electron transfer to the catalyst or the sacrificial agent, high stability under catalytic conditions, and are particularly suitable redox potentials for catalyst oxidation at all necessary oxidation stages.

**Figure 9:**
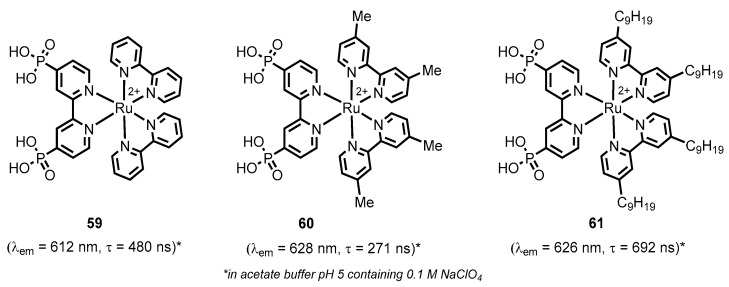
Chemical structures of ruthenium complexes **59**–**61**.

Mulyana et al. investigated four phosphorus-based ruthenium complexes, **59**–**61** and **63**, as photocatalysts for water decomposition (Figure 9, Scheme 4) [41]. The idea was to create a system in which Ru^2+^ cations are oxidized to Ru^3+^, while hydrogen ions are reduced to elemental hydrogen. Ru^2+^ cations can then be regenerated by the treatment with EDTA.

**Scheme 9:**
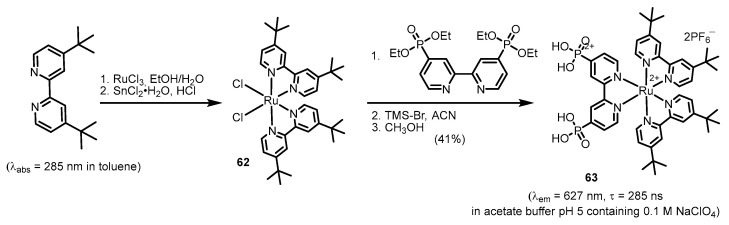
Synthesis of the complex **63**.

The authors prepared complexes **59**–**61** according to the previous reports. Complex **63** was synthesized by the addition of ruthenium(III) chloride to 4,4′-di-*t*-butyl-2,2′-bipyridine, followed by a reaction of complex **62** with a 4,4′-diethoxyphosphoryl-2,2′-bipyridine phosphonate ligand. Finally, phosphonate diesters were converted to the corresponding phosphonic acids **63** with TMS-Br (Scheme 9). The studies showed that **61** possessed the highest photocurrent and the highest excited state lifetime.

**Scheme 10:**
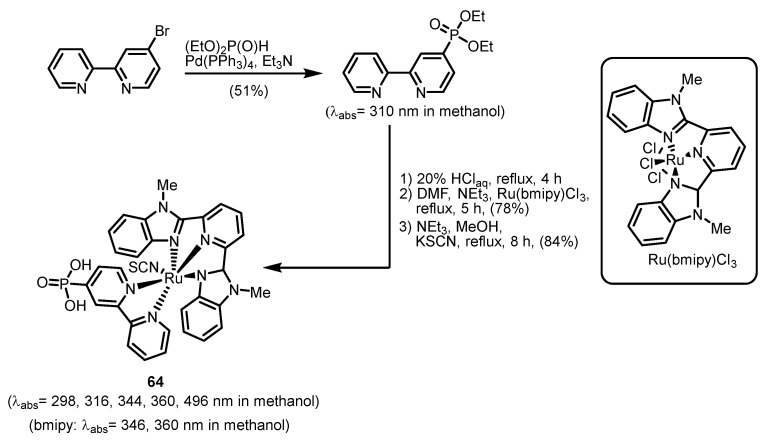
Synthesis of complex **64**.

Ruile and co-workers designed a set of Ru(II) complexes for photoelectrical devices based on TiO_2_. The authors synthesized the phosphonate ligand by coupling 4-bromo-2,2′-bipyridine with diethylphosphite, followed by hydrolysis with aqueous hydrochloric acid and an addition of potassium thiocyanate (Scheme 10) [42].

As it turned out, compound **64** existed in two isomeric forms, in which the phosphonic acid group was placed *cis* or *trans* with respect to the thiocyanate. It was proven that the isomer ratio was 1:6 (*cis* to *trans*). What is more, the authors showed that **64** sensitized TiO_2_ efficiently, but with lower incident monochromatic photon-electron conversion energy (IPCE). The phosphonic acid group made the complex less easily desorbed upon exposure to water, making it a perfect anchoring group.

**Figure 10:**
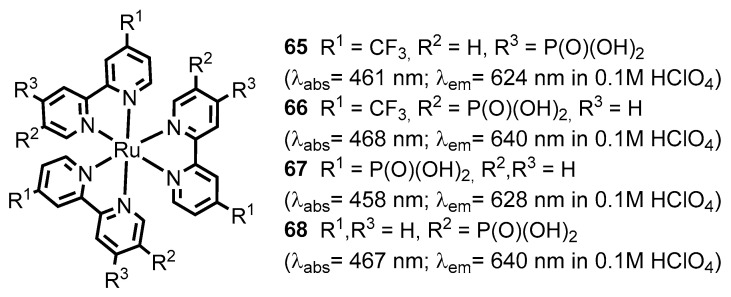
Chemical structures of complexes **65**–**68**.

Concepcion et al. synthesized a series of Ru(II) complexes, **65**–**68**, for application in artificial photosynthesis as oxidation catalysts (Figure 10) [43].

**Scheme 11:**
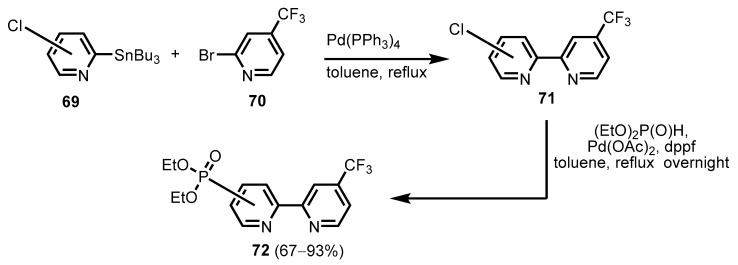
General synthesis of ligands **72**.

The synthesis of ligands **72** for **65**–**66** complexes had not been reported previously and it involved the Stille coupling of 2-bromo-pyridine **70** with the stannane **69**, followed by another coupling of the resulting product **71** with diethylphosphite (Scheme 11). All of the complexes exhibited absorption peaks at approximately 650 nm and 460 nm, and emission peaks near 630 nm. The former could be attributed to metal–ligand charge transfer. It was also found that the complexes were rather non-fluorescent, as their quantum yields varied from 0.0128 (for **68**) to 0.0379 (for **65**).

**Scheme 12:**
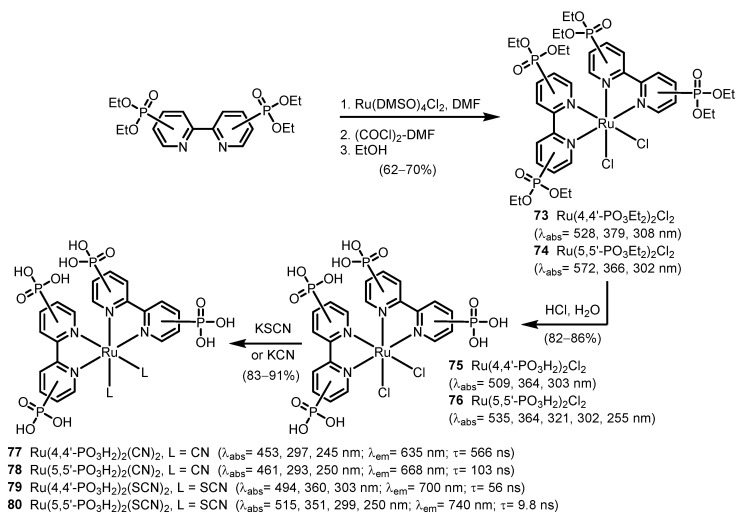
Synthesis and spectroscopic data of complexes **73**–**80** (absorption maximum measured in 0.1 N sulfuric acid; emission maximum measured for the anionic forms of the complexes at room temperature in deaerated methanol; emission lifetimes for the anionic forms of the complexes).

Bignozzi, Cano-Boquera et al. designed a strategy for the preparation of *cis* ruthenium complexes, which contained phosphonate moieties [44]. The authors hoped the complexes would be used in optoelectronics as photosensitizers. The synthesis involved the complexation of ruthenium(II) salt with bipyridine, yielding compounds **73** and **74** (Scheme 12).

The phosphonate complexes **73** and **74** were hydrolyzed to phosphonic acids **75** and **76**, and then chlorine atoms were replaced with cyanide or thiocyanide, giving compounds **77**–**80**. The NMR spectra showed that some protons of the bipyridine unit could be shifted as far as 10.1 ppm, due to the formation of the ring current circulation on pyridine. The spectroscopic measurements showed that complexes **73**–**80** exhibited blue-shift in the absorption spectrum, compared to analogous carbonyl compounds. Moreover, the authors studied these complexes as photosensitizers in solar cells, but compounds **73**–**80** showed less efficiency compared to carbonyl analogs. However, 4,4′-substituted bipyridyl complexes **73** and **77** revealed higher photoconversion efficiencies than 5,5′-substituted ones.

Bignozzi at al. studied osmium complexes containing a polypyridine ligand substituted by phosphonic acid groups **81** as sensitizers exhibiting photovoltaic performances. They demonstrated that complex tris(4,4′-bis(phosphonic acid)-2,2′-bipyridine)osmium(II) showed a higher red sensitivity with respect to the Ru(H_2_dcb)_2_(NCS)_2_ dye (H_2_dcb = 2,2′-bipyridine-4,4′-dicarboxylic acid), with incident monochromatic photocurrent conversion efficiency (IPCE) values of ca. 50% in the 600–700 nm spectral region. The complex showed characteristic absorption maxima at 310, 450 and 500 nm (in 0.1 N NaOH), emission at 750 nm (in MeOH) and its emission lifetime was 42.9 ns [45].

**Scheme 13:**
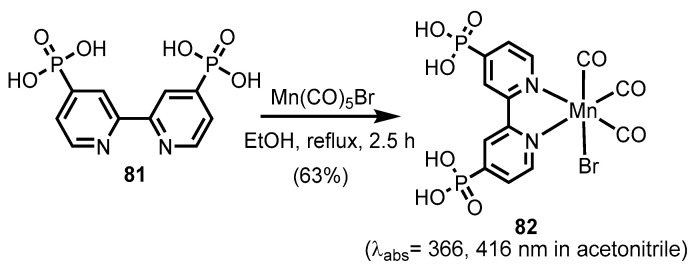
Synthesis of complex **82**.

Rosser, Windle and Reisner described the synthesis and spectral studies of Mn(II) complex **82** containing a phosphonic acid moiety (Scheme 13) [46]. Compound **82** contained only earth-abundant elements and could be used as a catalyst for the CO_2_ reduction to CO. The synthesis of **82** was accomplished by the complexation of pentacarbonyl manganese(I) bromide with 4,4′-bis(phosphonic acid)-2,2′-bipyridine **81**.

**Scheme 14:**
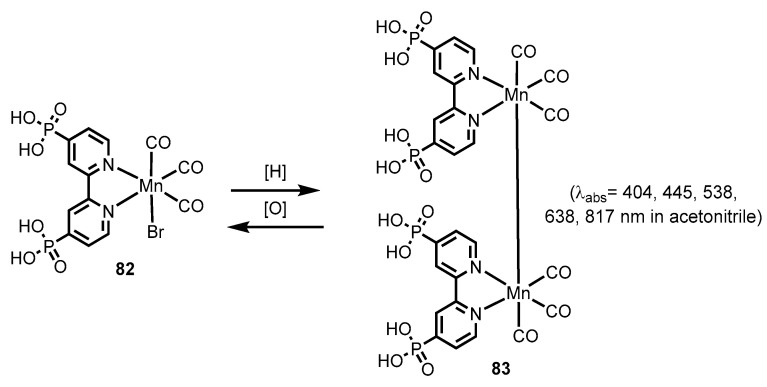
Synthesis of the dimer **83** and the regeneration of complex **82**.

The authors showed that the reduction of CO_2_ proceeded by the reduction of Mn(I) to the dimeric Mn(0) species **83**, which then reduced carbon dioxide with regeneration of **82** (Scheme 14).

FT-IR spectra have shown an increase in the intensity of absorption and the broadening of bands near 1950–2100 cm^−1^ upon the deposition of **82** on the TiO_2_ electrode. UV-Vis spectra have displayed bands near 400–500, 640 and 820 nm, which could be attributed to the formation of the Mn(0)-Mn(0) bond, which was not present before or after the reduction of CO_2_, thus confirming the proposed mechanism.

**Figure 11:**
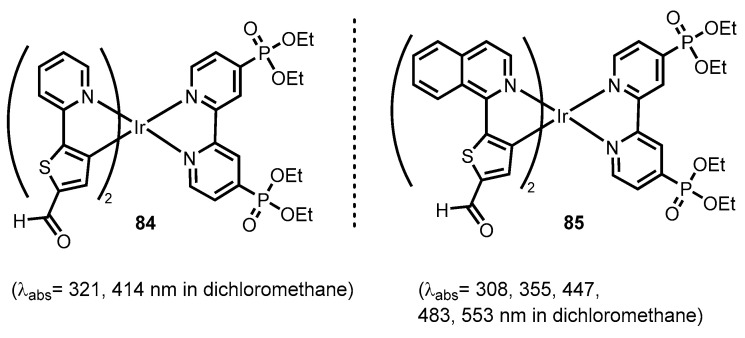
Chemical structures of iridium(III) complexes **84** and **85**.

Ho, Hannongbua and co-workers have investigated iridium(III) complexes **84** and **63** containing phosphonate moieties for optoelectronics (Figure 11) [47]. The authors tested various anchoring groups and concluded that phosphonate derivatives outperformed classical carbonyl anchors. The synthesis consisted of the substitution of IrCl_3_ with different ligands, which were prepared according to the literature procedures or bought from commercial sources.

The authors found that Ir-complex **85** exhibited more intensive and red-shifted light absorption than **84**, due to its extended conjugation. Furthermore, the authors tested **84** and **85** as photosensitizers in the light-driven hydrogen generation studies on Pt-TiO_2_ surfaces. It was shown that both compounds outclassed their carbonyl-containing counterparts, which could be attributed to better anchoring. Compound **85** gave the highest turnover value for this process.

**Figure 12:**
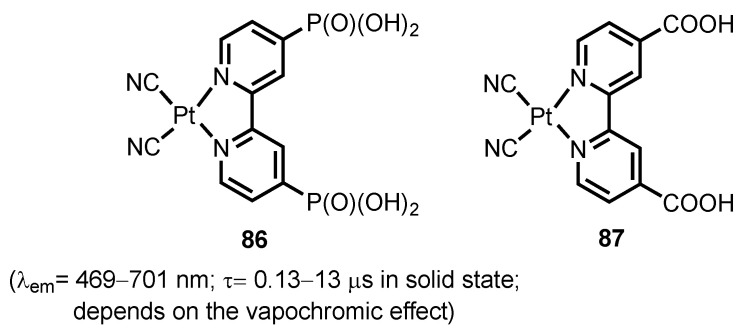
Chemical structures of platinum complexes **86** and **87**.

A luminescent Pt(II) complex, **86**, was successfully synthesized and its two-way vapochromic behavior was investigated (Figure 12) [48]. Emission spectroscopy revealed that the **86**·5H_2_O complex exhibited an orange emission at 585 nm that shifted in two directions, to a blue-green emission at 469 nm by drying at 100 °C, or to a red emission at 701 nm by drying under vacuum at room temperature. The comparable distances around the Pt(II) ions observed in **86** and **87** suggested that substitution of the carboxylic acid groups at the 4,4′-positions of the bpy ligand for phosphonic acid groups hardly affected the electronic state of the Pt(II) ion.

**Scheme 15:**
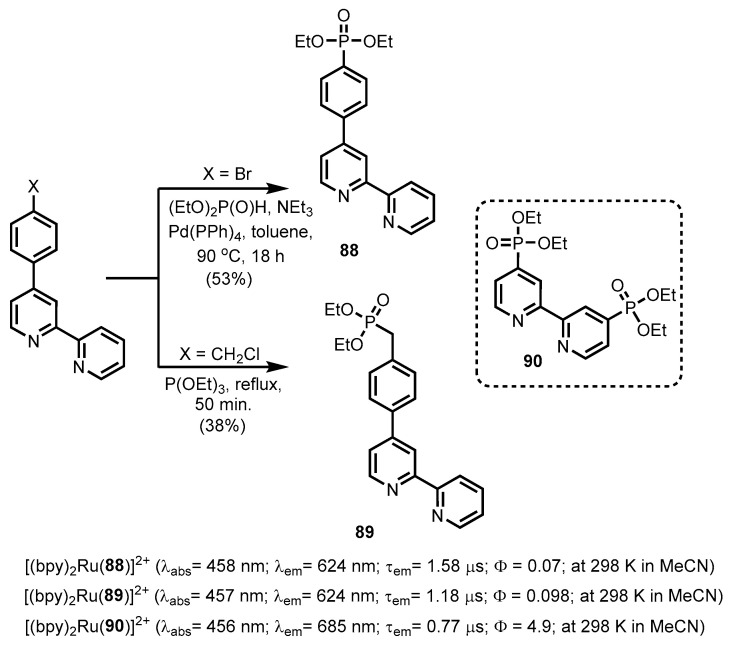
Synthesis and chemical structures of phosphonates **88**–**90**.

Schmehl and co-workers described the synthesis of bipyridyl complexes of Ru(II) for light-harvesting and -sensitization [49]. For this application, they synthesized six ligands: three phosphonates **88**–**90** and three corresponding phosphonic acids (Scheme 15). The synthesis of **88** involved the coupling of aryl bromide with diethylphosphite, while phosphonate **89** was obtained by the Arbuzov reaction.

Ru(II) complexes of ligands **88**–**90** showed luminescence at room and low temperatures, with moderate lifetimes. The authors showed that [(bpy)_2_Ru(**90**)]^2+^ exhibited a significant blue-shift in the emission spectrum upon hydrolysis to the corresponding acid. The effect was attributed to the large difference in the electron-accepting ability between **90** and its corresponding acid. It was also shown that the luminescence of phosphonic acid complexes could be quenched by the addition of Cu(II) or Fe(III) ions in non-aqueous solvents.

**Figure 13:**
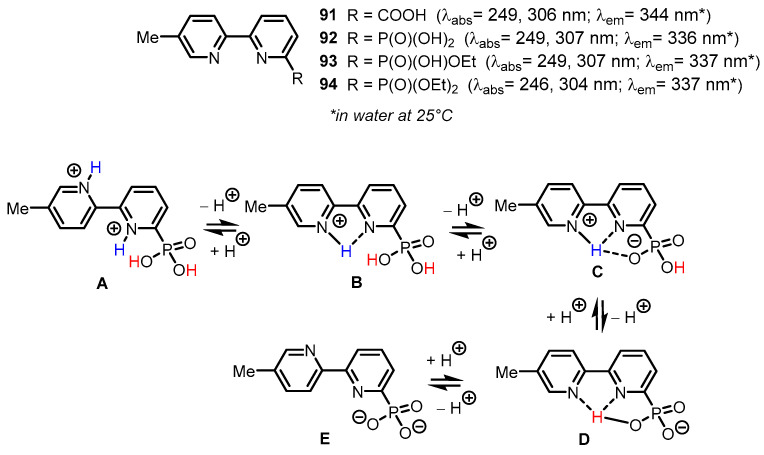
Chemical structures of ligands **91**–**94** and equilibrium **A**–**D** of **92** at different pH.

A series of four ligands, based on a 5′-methyl-2,2′-bipyridyl framework substituted in the 6 positions of **91**, **92**, **93** and **94**, were described by Ziessel et al. (Figure 13) [50]. As was shown, the lanthanide complexes of the phosphonic acid derivatives were up to two orders of magnitude more stable than those of the carboxylic acid ligand. The absorption spectra of the ligands featured two main bands (with additional shoulders) located around 250 nm and 305 nm, and assigned to π→π* transitions, mainly located on the pyridine units for the latter, while the former contained substantial contributions from the carboxylic (**91**) or phosphoryl groups (**92**–**94**). The ligand-centered luminescence of the studied complexes displayed essentially the same features as the free ligand luminescence.

The deprotonation of the bipy core of **92** at the pH range of 1.21–8.03 was not accompanied by the classical hypsochromic shift in the UV-Vis absorption spectra from 308 nm to 290 nm, which was usually associated with the *cis*−*trans* isomerization. The authors explained the unusual behavior by the presence of the phosphonate group, which kept stabilizing the *cis* form **D** by intramolecular hydrogen bonding, thereby maintaining the corresponding π→π* transitions at low energies. Only when the last proton was removed at higher pH values (**E**) did the nitrogen lone pair repulsion become large enough to induce the hypsochromic shift. Similar behavior was not observed for the other ligands as no H-bonding interaction could stabilize the non-protonated bipy subunit.

#### 2.2.3. Terpyridinephosphonic Acids and Derivatives

**Figure 14:**
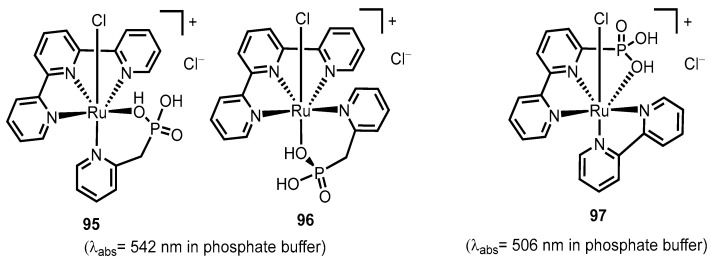
Chemical structures of complexes **95**–**97**.

UV-visible spectra were obtained for **95**–**97** as a function of pH, and these data suggested that the observed pK_a_s corresponded to deprotonation of a bound water molecule, for example [Ru(bpyPO_3_)(bpy)(OH_2_)]→[Ru(bpyPO_3_)(bpy)(OH)]^−^, indicating that the bound chloride ions in **95**, **96** and **97** were displaced by aqua ligands upon dissolution (Figure 14) [51]. Each compound was found to exhibit proton-coupled electron transfer (PCET), and under aqueous conditions **97** oxidized secondary C−H bonds using sodium periodate (NaIO_4_) as the primary oxidant.

**Scheme 16:**
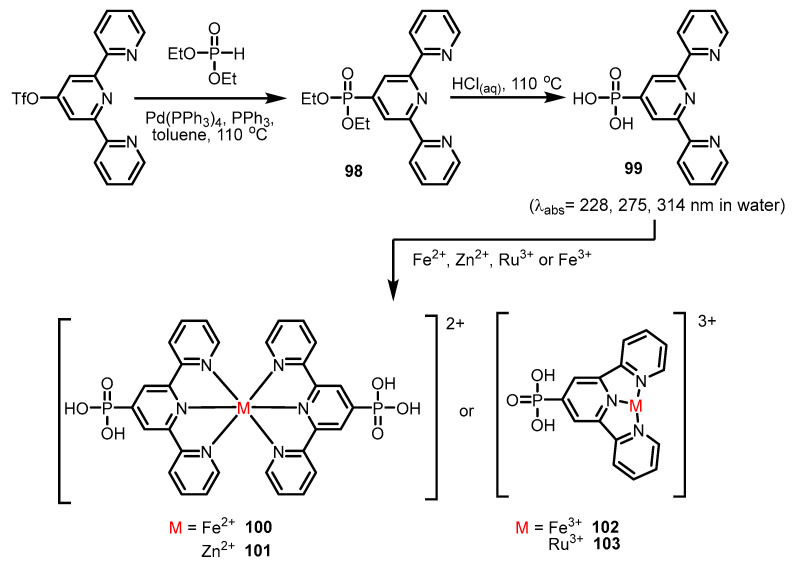
The synthesis of terpyridyl ligand **99** for the identification of ions Fe(II), Fe(III), Ru(III) and Zn(II) in dilute aqueous solutions.

Zenkina et al. synthesized a terpyridyl ester, **98,** and acid **99** which was used as a ligand in Fe^2+^, Fe^3+^, Ru^3+^ and Zn^2+^ complexes (Scheme 16) [10]. They aimed to obtain a sensor that could be used in logic gates to detect these ions in concentrations as low as parts per billion. As shown in Scheme 16, 4′-{[(trifluoromethyl)sulfonyl]oxy}-2,2′:6′,2″-terpyridine was coupled with diethylphosphite in the presence of tetrakis(triphenylphosphine)palladium(0), producing the phosphonate derivative **98**. The phosphonic acid ligand **99** was obtained by treating the corresponding ester with a diluted aqueous solution of hydrochloric acid. The authors added the ligand to the solutions of corresponding metal cations, yielding complexes **100**–**103**, however, only structure **100** has been crystallographically determined.

As typical for such structures, the metal–ligand absorption bands were observed. At 290 nm, only complexes **102** and **103** showed absorption, while at 400 nm only complexes **100** and **102** absorbed light. At 560 nm, only **100** showed absorption. The detection of **102** was possible due to strong fluorescence at 360 nm upon excitation at 326 nm. Thus, it was possible to simultaneously differentiate and measure concentrations of Fe^2+^, Zn^2+^, Ru^3+^ and Fe^3+^ in the presence of ligand **99**.

**Scheme 17:**
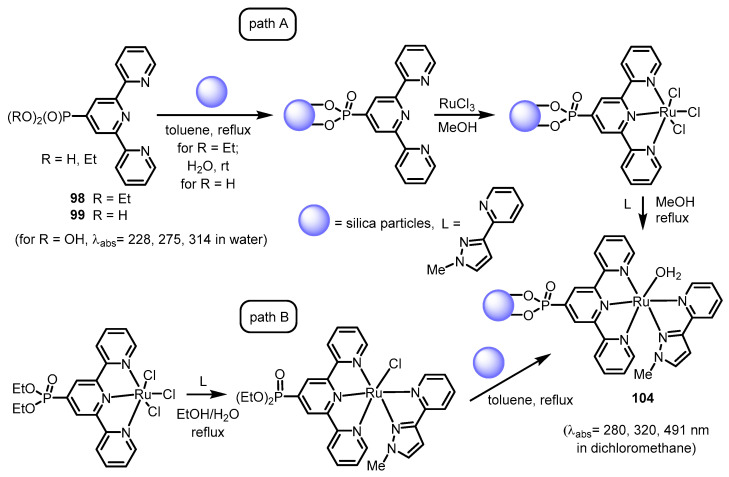
Synthetic strategies for the synthesis of Ru complex **104**.

A molecular ruthenium aqua complex, **104**, was synthesised from terpyridinephosphonic ester **98** or acid **99**, and grafted by phosphonate group to mesoporous silica and silica-coated magnetic particles onto two independent paths A and B (Scheme 17) [52]. The UV-Vis spectra of **98** exhibited ligand-based π→π* bands below 350 nm and relatively intense bands above 350 nm, mainly due to dπ(Ru)→π*(L) metal-to-ligand charge-transfer transitions, similar to those obtained for homogeneous complex, which indicated that electronic properties remained unchanged. The catalytic performance and the reutilization of these hybrid materials were explored in the epoxidation of alkenes with PhI(OAc)_2_.

**Scheme 18:**
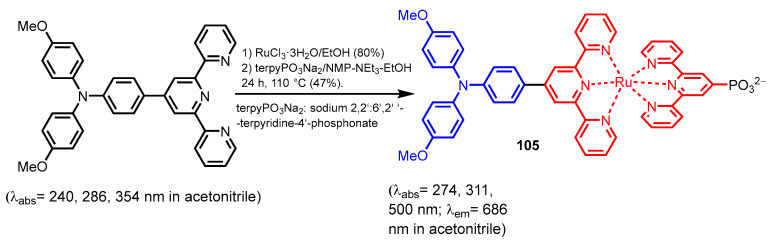
Synthesis of complex sensitizer/donor type **105**.

**Figure 15:**
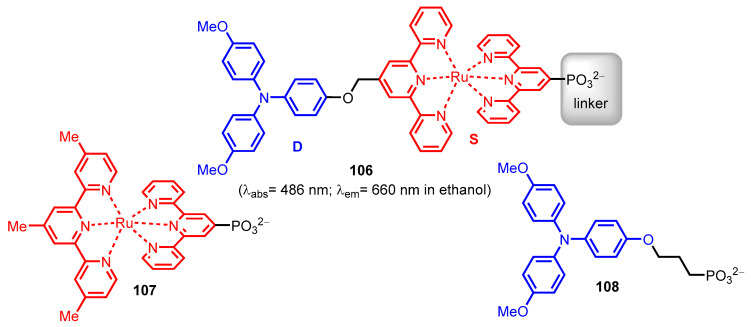
Chemical structures of the sensitizer/donor **106** and the dyad **107**/**108**.

**Figure 16:**
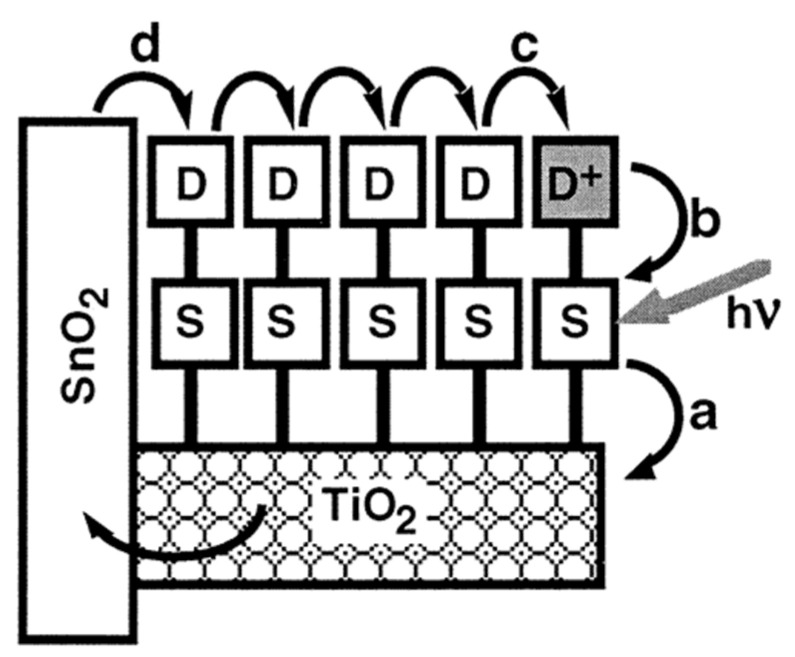
Proposed electron fluxes in the illuminated heterotriad **106**|TiO_2_ on SnO_2_ (Reprinted with permission from [53]).

The photoinduced charge separation in three different assemblies composed of an electron donor D and based on ligand **99** chromophore sensitizer S adsorbed on nanocrystalline TiO_2_ films by a phosphonate group to efficiently convert light into electrical energy was investigated by Bonhôte et al. (Scheme 18, Figure 15) [53]. In all of the systems, the sensitizer was a ruthenium(II) bis-terpyridine complex and the donor was a 4-(*N,N*-di-*p*-anisylamino) phenyl group linked to the 4′ position of the terpyridine, either directly (**105**) or via a benzyl ether interlocking group (**106**). In the third system, sensitizer **107** and donor **108** were co-adsorbed on the surface. Absorption spectra of heterotriads **105**|TiO_2_, **106**|TiO_2,_ and **107**,**108**|TiO_2_ under positive polarization (0.5 V), after 10 min. illumination, showed a new strong absorption band with a maximum in the range of 730–750 nm. Light-induced excitation of the sensitizer S was followed by electron injection (a, Figure 16) into the TiO_2_ and oxidation (b) of the donor D. Lateral conduction (c) inside the monolayer allowed electrons to flow from the SnO_2_ (d) to the oxidized donors.

#### 2.2.4. Phenantrolinephosphonic Acids and Derivatives

**Scheme 19:**
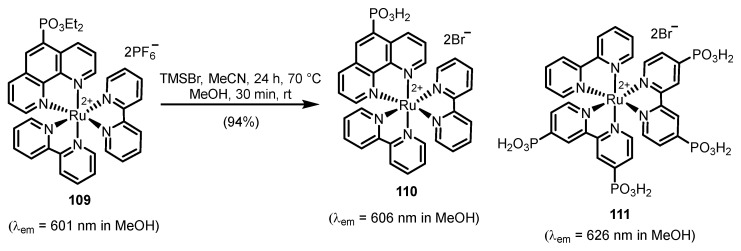
Synthesis of complex **110**.

Complexes with a phosphonate group in the five-position of the phenanthroline ligand have also been described by Rau et al. [54]. As it turned out, the substitution pattern used only slightly changed photophysical properties of the compounds, compared to the reference molecule **109** in the aqueous medium (Scheme 19). However, conversion of diethyl phosphonate ester in **109** to the corresponding phosphonic acid **110** drastically reduced photostability in water. The water oxidation catalytic experiments of compound **110** showed the best performance. **111** demonstrated very poor catalytic performance, although it had good stability and higher oxidation potential compared to **110**. The cationic photosensitizers **109** and [Ru(bpy)_3_](PF_6_)_2_ were unable to provide any photocatalytic activity.

**Figure 17:**
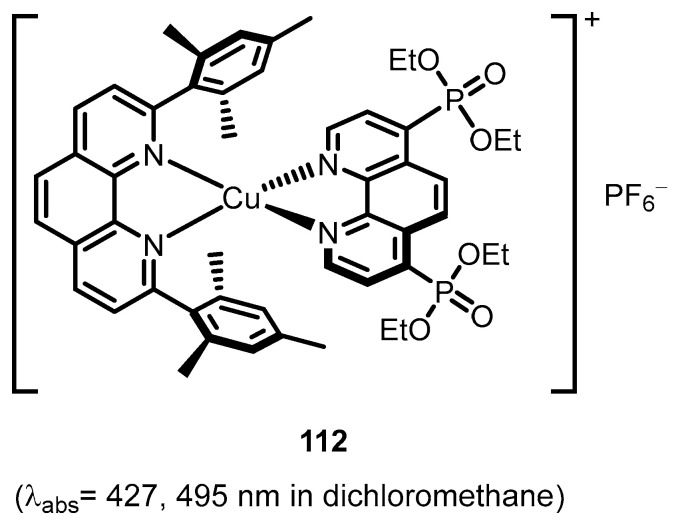
Chemical structure of complex **112**.

Mulfort and co-workers designed a series of Cu(I) phenantroline complexes including hexafluorophosphate complex **112** (Figure 17) [55]. The goal was to study the directionality of metal–ligand charge transfer in these complexes to further understand the localization of electronic excited states. The authors were able to show that **112** shifted the localization the most towards the phosphorus-containing ring.

The ligands were synthesized according to previously reported procedures—the synthesis involved the coupling of 4,7-dibromo-1,10-phenanthroline with triethyl phosphite. Compound **112** contained the most electron-withdrawing group in the investigated complexes, which made the absorption peak the most red-shifted to approximately 500 nm.

#### 2.2.5. Pyrazine-, Pyrazole- and Phenoxazinonephosphonic Acids and Derivatives

**Figure 18:**
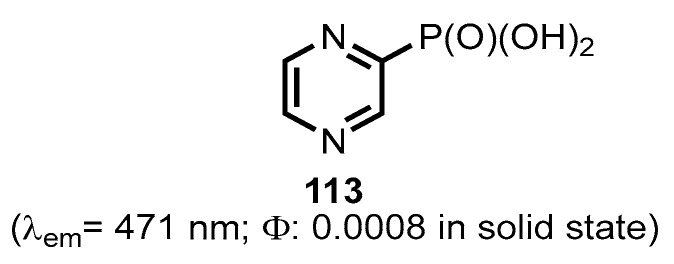
Chemical structure of 2-pyrazinephosphonic acid **113**.

Ma, Yuan et al. described crystal structures of new Zn(II) and Cd(II) complexes of 2-pyrazinephosphonic acid **113** (Figure 18) [56]. The complexes were synthesized by the refluxing of ZnCl_2_ or CdCl_2_·2.5H_2_O, 2-pyrazinephosphonic acid in water solution. The obtained complexes were Zn(C_4_H_3_N_2_PO_3_) (**114**), Cd[(C_4_H_3_N_2_PO_3_)(H_2_O)] (**115**) and Cd[(C_4_H_3_N_2_PO_3_Cl]·H_2_O (**116**) (upon addition of HCl to the refluxed mixture).

The fluorescence emission peaks for compounds **114**–**116** were approximately the same as for 2-pyrazinephosphonic acid, all observed near 470 nm. However, the authors noticed that the emission intensity increased with increased rigidity of 2-pyrazinephosphonic acid in forming the co-ordination polymers. Thus, the fluorescence intensity increased in the series: **113** < **114** < **115** < **116**. It was also shown that **116** had the highest quantum yield of 1.55%.

**Figure 19:**
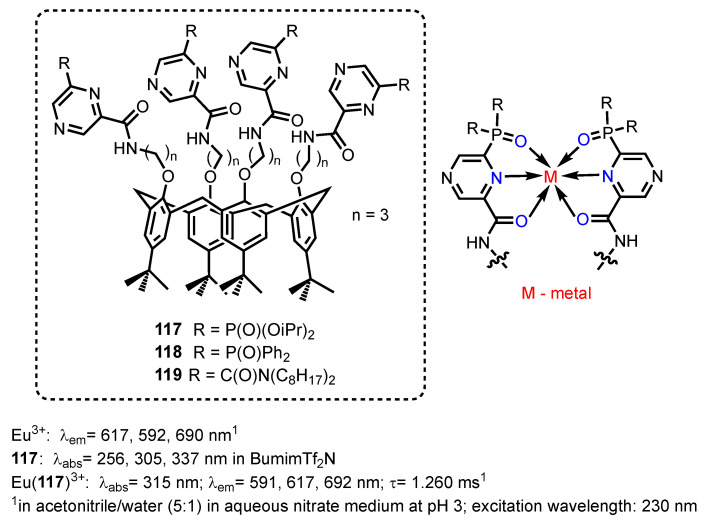
Chemical structures of pyrazine-functionalized calix [4]arene ligands **117**–**119**, and a schematic presentation of metal co-ordination.

**Figure 20:**
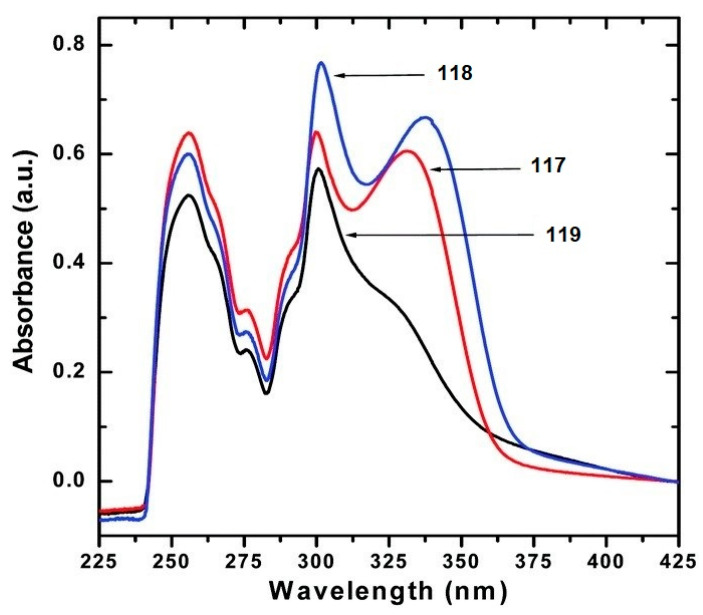
Absorption spectra of ligands **117**–**119** in BumimTf_2_N (conc.: 0.25 mmol/L at 25 °C) (reprinted with permission from [58], Copyright The Royal Society of Chemistry 2015).

Heteroaromatic compounds with phosphoryl group, such as **117** and **118**, or the amide group **119**, when deposited on a stable and defined calixarene structure, acted as ligands complexing lanthanides and actinides (Figure 19) [57,58]. The analysis using ligand solutions of different concentrations indicated that, for all three ligands, the absorption bands were in the wavelength region of 285 nm–400 nm, followed Beer’s Law and were suitable for spectrophotometry (Figure 20).

All three ligands formed the 1:1 metal-ligand complex with Eu^3+^, and the stability constants followed the order: **119** < **117** < **118**. This trend could be interpreted by the difference in the basicity of the functional groups and the steric effects of the ligands. Two pendant arms of the ligand co-ordinate to Eu^3+^ in a tridentate mode, and the other two arms were either non-bonded or very weakly bonded with the metal ion (Figure 19).

**Scheme 20:**
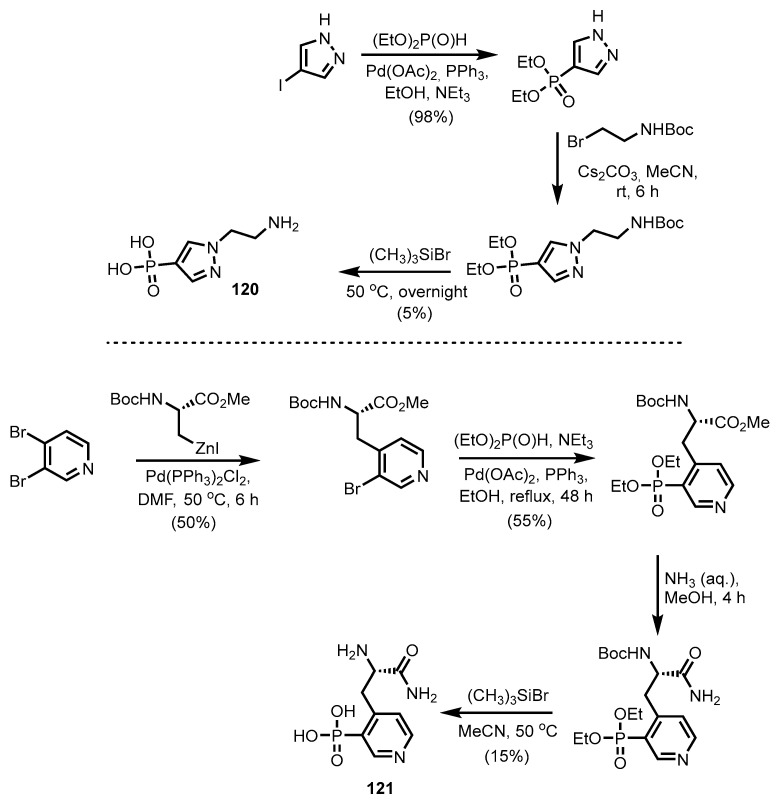
Synthesis of the phosphopyrazole **120** and the pyridyl amino amide **121**.

Jackson, Muimo et al. designed and synthesized analogs of τ- and π-phosphohistidine (phosphopyrazole **120** and pyridyl amino amide **121**), which were used to generate polyclonal antibodies (Scheme 20) [59]. The compounds were subsequently tested in immunological studies concerning the selectivity of the antibodies produced by **120** and **121**. The synthesis of both compounds involved the same key steps, namely the introduction of the diethoxyphosphoryl group by coupling (EtO)_2_P(O)H with the corresponding aryl bromide, followed by hydrolysis of the phosphonate ester with trimethylsilyl bromide to form phosphonic acids **120** and **121**. Furthermore, immunofluorescence studies have shown that **120**–**121**-induced antibodies were present throughout human bronchial epithelial cells, as well as in human neurons, with their higher concentration near the nucleus.

**Scheme 21:**
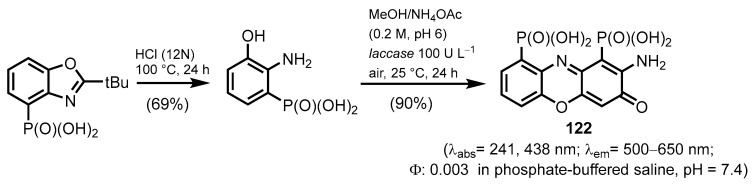
Synthesis of the aminophenoxazinone **122**.

A report on a series of water-soluble aminophenoxazines that were evaluated for vital imaging in cell biology using an enzymatic synthetic strategy as an alternative to the nitrosative or chemical oxidative coupling of phenolic precursors was synthesized by Brynaert et al. [60]. Thus, the 1,9-bis-phosphorylated aminophenoxazinone dye **122** was synthesized in 90% yield by taking advantage of laccase-enzyme-mediated oxidation in water (Scheme 21). The UV-Vis spectra of compound **122** showed absorption at 241 and 438 nm, with a high *ε* value of 8.8 × 10^3^ M^−1^ cm^−1^. However, the fluorescence quenching observed when **122** was mixed with Mg^2+^ and/or Ca^2+^ brought about a heavy handicap in the cell labeling application. On the other hand, this could lead to its development as a sensor for variations in divalent cation concentrations in the high range.

#### 2.2.6. Carbazolephosphonic Acids and Derivatives

**Figure 21:**
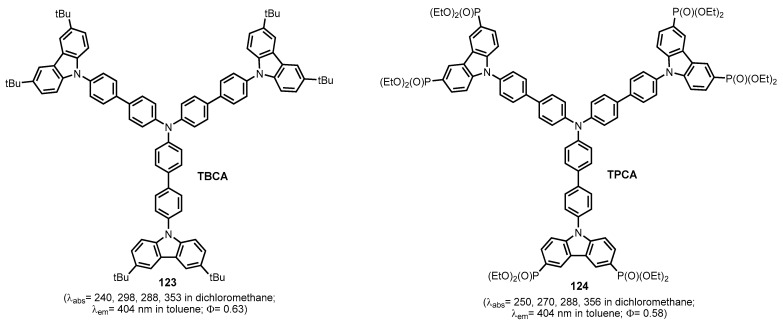
Chemical structures of TBCA (**123**) and TPCA (**124**).

A solution-processable, deep-blue fluorescent emitter, **124** (TPCA), and its optical properties were compared with prototype **123** (TBCA) (Figure 21) [61]. The identical optical band gap demonstrated that the electronic structure of the excited state was independent of the attached surface groups. In addition, TPCA in the film showed a red-shift of 8 nm compared to TBCA, due to a solvation effect. The corresponding non-doped, single-layer SMOLED with TPCA showed a peak current efficiency about three orders of magnitude higher than that of the prototype with *tert*-butyl substituents.

**Scheme 22:**
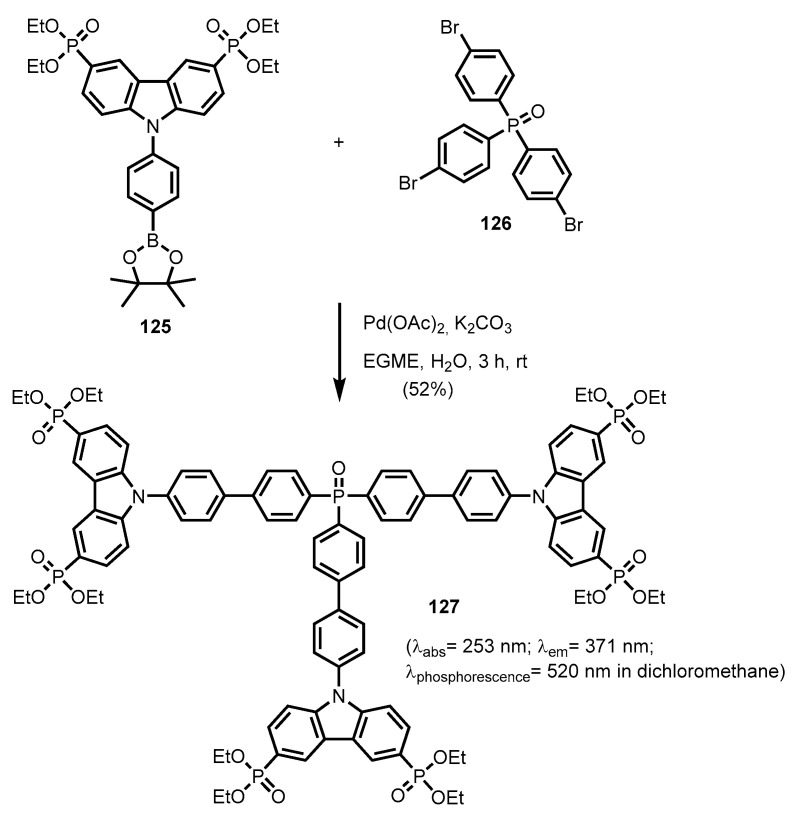
Synthesis of phosphonate **127** (TPPO).

Ding and co-workers reported the application of aryl phosphine oxide and phosphonate as an electron injection layer (EIL) used in polymer light-emitting diodes (PLEDs) [62]. The aryl phosphine oxide displayed high conductivity, while the phosphonate **127** (TPPO) was a good electron injector. Thus, the EIL inherited both of these properties. The synthesis of phosphonate **127** is especially worth attention. It involved the Suzuki coupling between the pinacol boronic ester **125** and tri-(*p*-bromophenyl)phosphine oxide **126** in the presence of palladium(II) acetate and potassium carbonate in a mixture of ethylene glycol monomethyl (EGME) and water (Scheme 22) [63]. As reported, the optical band gap for **127** was 3.49 eV. The absorption peak was observed at 250 nm, while the emission fell at 370 nm. The authors also recorded the phosphorescence spectrum at low temperatures with the maximum observed at approximately 520 nm. It was concluded that phosphonate **127** could be used as an electron injector in PLED devices. Moreover, such an application would be favorable if one desired to reduce the possibility of luminescence-quenching by energy transfer from an emitter to **127**.

**Figure 22:**
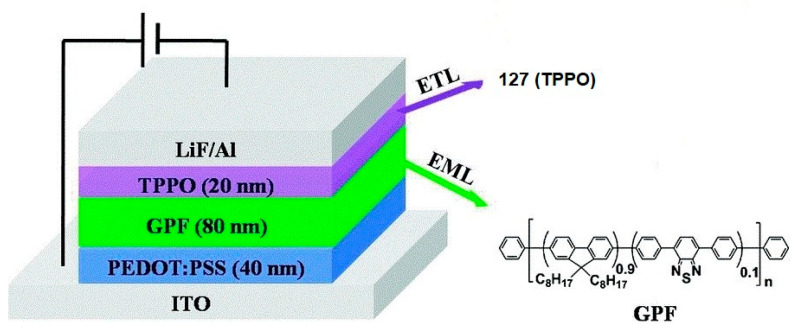
Device configuration of multilayer PLEDs and molecular structures of used materials (GPF—green-emitting polyfluorene; PEDOT:PSS—poly(3,4-ethylenedioxythiophene)-poly(styrenesulfonate); ETL—electron-transporting layer; EML—emitting layer; ITO—indium tin oxide) (Used with permission of The Royal Society of Chemistry, from [63], Copyright 2016; permission conveyed through Copyright Clearance Center, Inc.).

The same authors also used compounds **124** and **127** for the passivation of perovskite quantum dots in PeQLEDs, compensating for lead defects, common in such structures (Figure 22, Scheme 22) [64]. The authors found that the P=O bond co-ordinated the exposed Pb atoms, significantly improving photoluminescence quantum yields. It was also proven that the usage of **124** in such systems was slightly advantageous to **127** since **124** could be dispersed more easily. Photoluminescence spectra of the perovskite quantum dots used, and those doped with **127** or **124,** showed an emission peak at 518 nm, indicating that neither the amine nor the phosphine oxide changed the emission. Moreover, the lifetimes increased by 10 or 5 ns (**124** and **127,** respectively), indicating that the non-radiative exciton decay was suppressed.

Phosphonate-functionalized triaryl phosphine oxide **127** (TPPO) has been used for the successful fabrication of high-performance, solution-processed multilayer PLEDs (Figure 22) [63]. According to the absorption onset of **127**, the optical band gap was estimated to be 3.49 eV, and the corresponding emission fell into the ultraviolet region with a peak at 371 nm. The phosphorescent spectrum of **127** in toluene was recorded at 77 K and the calculated triplet energy level was about 2.65 eV, which was mainly determined by the biphenyl moiety. This suggested that **127** might also be suitable for green and red phosphorescent devices.

It was found that the inclusion of an additional TPPO layer resulted in an approximately 16-fold increase in the device performance compared to a control single-layer device without **127**.

## 3. Conclusions

This review, covering the period of 1994–2024, discussed methods for the synthesis and applications of heteroarylphosphonates and their complexes with metals utilizing their optical properties. Heteroaryl moieties of the described compounds contain nitrogen and sulfur atoms in the ring, but the vast majority involve pyridine derivatives, and they are often associated with the easy availability of these derivatives and their good complex-forming properties. The introduction of phosphonate groups makes it possible to manipulate the electronic and optical properties of the described compounds, but also to anchor them on metal oxide surfaces for device applications. In the construction of optoelectronic devices based on D-π-A structure, the presence of phosphonate groups ensures good solubility and the ability to produce good-quality, single layers in wet processes. This can improve the electron injection and transporting capability. The lack of a major influence on the emission wavelength and photoluminescence quantum yield of the molecule is also of great importance in this case. The heteroarylphosphonates are also widely used in co-ordination chemistry as ligands in complexes with lanthanides and actinides, among others, for separating radioactive waste. In this case, their fluorescent properties enable the identification of the formed compounds. Moreover, phosphonate functions are preferred for their high affinity toward metal ions compared to their carboxylated counterparts, and for their steric hindrance that favors the formation of less-hydrated complexes.

The advances included in this review will open a window to the further development of heteroarylphosphonates with optical properties, whose potential has still not been sufficiently explored. This can be attempted by expanding the range of heteroatoms and metals used, extending the length of the aromatic conjugation, or finally changing the position of the phosphonate substituents in the heteroaromatic ring. These changes, however, will often involve the need to develop new methods of synthesizing these compounds, giving organic chemists an impetus to act.

## Data Availability

No new data were created or analyzed in this study.

## References

[1] Bałczewski P., Owsianik K., Higham L.J., Allen D.W. (2024). Quinquevalent phosphorus acids. Organophosphorus Chemistry: Volume 52.

[2] Koprowski M., Owsianik K., Knopik Ł., Vivek V., Romaniuk A., Różycka-Sokołowska E., Bałczewski P. (2022). Comprehensive Review on Synthesis, Properties, and Applications of Phosphorus (PIII, PIV, PV) Substituted Acenes with More Than Two Fused Benzene Rings. Molecules.

[3] Khalkhali F.S., Kowsari E., Ramakrishna S., Eqbalpour M., Gheibi M., Esmaili H. (2024). A review on the photosensitizers used for enhancing the photoelectrochemical performance of hydrogen production with emphasis on a novel toxicity assessment framework. Int. J. Hydrogen Energy.

[4] Charpentier C., Salaam J., Nonat A., Carniato F., Jeannin O., Brandariz I., Esteban-Gomez D., Platas-Iglesias C., Charbonnière L.J., Botta M. (2020). pH-Dependent Hydration Change in a Gd-Based MRI Contrast Agent with a Phosphonated Ligand. Chem. Eur. J..

[5] Pan Y., Li W., Hui Q., Yang Z., Barhoum A., Altintas Z. (2023). Chapter 4—Biosensors for bacteria detection. Advanced Sensor Technology.

[6] Hasegawa M., Ohmagari H., Tanaka H., Machida K. (2022). Luminescence of lanthanide complexes: From fundamental to prospective approaches related to water- and molecular-stimuli. J. Photochem. Photobiol. C Photochem. Rev..

[7] Virender, Chauhan A., Kumar A., Singh G., Solovev A.A., Xiong J., Liu X., Mohan B. (2024). Photonic properties and applications of multi-functional organo-lanthanide complexes: Recent advances. J. Rare Earths.

[8] Arya M., Heera S., Meenu P., Deepa K.G. (2024). Organic-inorganic hybrid materials and architectures in optoelectronic devices: Recent advancements. ChemPhysMater.

[9] Mauri L., Colombo A., Dragonetti C., Roberto D., Fagnani F. (2021). Recent Investigations on Thiocyanate-Free Ruthenium(II) 2,2′-Bipyridyl Complexes for Dye-Sensitized Solar Cells. Molecules.

[10] Laschuk N.O., Ebralidze I.I., Spasyuk D., Zenkina O.V. (2016). Multi-Readout Logic Gate for the Selective Detection of Metal Ions at the Parts Per Billion Level. Eur. J. Inorg. Chem..

[11] Duan T., Chen Q., Hu D., Lv J., Yu D., Li G., Lu S. (2022). Oligothiophene-based photovoltaic materials for organic solar cells: Rise, plateau, and revival. Trends Chem..

[12] Iftikhar R., Khan F.Z., Naeem N. (2024). Recent synthetic strategies of small heterocyclic organic molecules with optoelectronic applications: A review. Mol. Divers..

[13] Bair J.S., Harrison R.G. (2007). Synthesis and Optical Properties of Bifunctional Thiophene Molecules Coordinated to Ruthenium. J. Org. Chem..

[14] Freeman J.L., Zhao Q., Zhang Y., Wang J., Lawson C.M., Gray G.M. (2013). Synthesis, linear and nonlinear optical properties of phosphonato-substituted bithiophenes derived from 2,2′-biphenol. Dalton Trans..

[15] Freeman J.L., French D.N., Zhao Q., Wang J., Zhang Y., Hamilton T.P., Lawson C.M., Gray G.M. (2017). Synthesis, Linear, and Nonlinear Optical Properties of Phosphonato-Substituted Bithiophenes Derived from 2,2-Dimethyl-1,3-propanediol. Eur. J. Inorg. Chem..

[16] Horne J.C., Blanchard G.J. (1998). The Role of Substrate Identity in Determining Monolayer Motional Relaxation Dynamics. J. Am. Chem. Soc..

[17] Byrd H., Whipps S., Pike J.K., Ma J., Nagler S.E., Talham D.R. (1994). Role of the template layer in organizing self-assembled films: Zirconium phosphonate monolayers and multilayers at a Langmuir-Blodgett template. J. Am. Chem. Soc..

[18] Lo C.-Y., Chen C.-H., Tsai T.W.T., Zhang L., Lim T.-S., Fann W., Chan J.C.C., Luh T.-Y. (2010). Efficient Energy and Electron Transfer between Donor and Acceptor Chromophores in Aluminophosphate Hybrid Materials. J. Chin. Chem. Soc..

[19] Mulhern K.R., Orchard A., Watson D.F., Detty M.R. (2012). Influence of Surface-Attachment Functionality on the Aggregation, Persistence, and Electron-Transfer Reactivity of Chalcogenorhodamine Dyes on TiO_2_. Langmuir.

[20] Sabatini R.P., Mark M.F., Mark D.J., Kryman M.W., Hill J.E., Brennessel W.W., Detty M.R., Eisenberg R., McCamant D.W. (2016). A comparative study of the photophysics of phenyl, thienyl, and chalcogen substituted rhodamine dyes. Photochem. Photobiol. Sci..

[21] Sheokand M., Rout Y., Misra R. (2022). Recent development of pyridine based charge transporting materials for organic light-emitting diodes and perovskite solar cells. J. Mater. Chem. C.

[22] Saeed A., Faisal M., Larik Ali F., El-Seedi R.H., Channar Ali P., Shahzad D. (2018). Recent Progress in Pyridine Containing Heterocycles as High Performance Host Materials for Blue PHOLEDs. Mini-Rev. Org. Chem..

[23] dos Santos P.L., Chen D., Rajamalli P., Matulaitis T., Cordes D.B., Slawin A.M.Z., Jacquemin D., Zysman-Colman E., Samuel I.D.W. (2019). Use of Pyrimidine and Pyrazine Bridges as a Design Strategy To Improve the Performance of Thermally Activated Delayed Fluorescence Organic Light Emitting Diodes. ACS Appl. Mater. Interfaces.

[24] Chaudhran P.A., Sharma A. (2022). Progress in the Development of Imidazopyridine-Based Fluorescent Probes for Diverse Applications. Crit. Rev. Anal. Chem..

[25] Zhang J., Jiang Y., Cheng X., Xie Y., Zhao J., Weng J. (2023). Recent advances in versatile pyridazine-cored materials: Principles, applications, and challenges. J. Mater. Chem. C.

[26] Ng X.Y., Fong K.W., Kiew L.V., Chung P.Y., Liew Y.K., Delsuc N., Zulkefeli M., Low M.L. (2024). Ruthenium(II) polypyridyl complexes as emerging photosensitisers for antibacterial photodynamic therapy. J. Inorg. Biochem..

[27] Fry J.A., Samanamu C.R., Montchamp J.-L., Richards A.F. (2008). A Mild Synthetic Route to Zinc, Cadmium, and Silver Polymers with (2-Pyridyl)phosphonic Acid: Synthesis and Analysis. Eur. J. Inorg. Chem..

[28] Chen R., Chen Y., Zeng X., Li J., Luo X. (2017). Transition metal phosphonates with pyridyl-based phosphonic acids: Synthesis, characterization and luminescence properties. J. Coord. Chem..

[29] Salaam J., Tabti L., Bahamyirou S., Lecointre A., Hernandez Alba O., Jeannin O., Camerel F., Cianférani S., Bentouhami E., Nonat A.M. (2018). Formation of Mono- and Polynuclear Luminescent Lanthanide Complexes based on the Coordination of Preorganized Phosphonated Pyridines. Inorg. Chem..

[30] Hamon N., Godec L., Jourdain E., Lucio-Martínez F., Platas-Iglesias C., Beyler M., Charbonnière L.J., Tripier R. (2023). Synthesis and Photophysical Properties of Lanthanide Pyridinylphosphonic Tacn and Pyclen Derivatives: From Mononuclear Complexes to Supramolecular Heteronuclear Assemblies. Inorg. Chem..

[31] Knighton R.C., Soro L.K., Troadec T., Mazan V., Nonat A.M., Elhabiri M., Saffon-Merceron N., Djenad S., Tripier R., Charbonnière L.J. (2020). Formation of Heteropolynuclear Lanthanide Complexes Using Macrocyclic Phosphonated Cyclam-Based Ligands. Inorg. Chem..

[32] Zeng D., Ren M., Bao S.-S., Li L., Zheng L.-M. (2014). A luminescent heptanuclear DyIr6 complex showing field-induced slow magnetization relaxation. Chem. Commun..

[33] Zeng D., Yuan X.-A., Liu J.-C., Li L., Wang L.-P., Qin M.-F., Bao S.-S., Ma J., Zheng L.-M. (2019). Cyclometalated Iridium(III) Complexes Incorporating Aromatic Phosphonate Ligands: Syntheses, Structures, and Tunable Optical Properties. ACS Omega.

[34] Lawson T., Gentleman A.S., Pinnell J., Eisenschmidt A., Antón-García D., Frosz M.H., Reisner E., Euser T.G. (2023). In situ Detection of Cobaloxime Intermediates During Photocatalysis Using Hollow-Core Photonic Crystal Fiber Microreactors. Angew. Chem. Int. Ed..

[35] Lakadamyali F., Reisner E. (2011). Photocatalytic H2 evolution from neutral water with a molecular cobalt catalyst on a dye-sensitised TiO2 nanoparticle. Chem. Commun..

[36] Chauvin A.-S., Comby S., Baud M., De Piano C., Duhot C., Bünzli J.-C.G. (2009). Luminescent Lanthanide Helicates Self-Assembled from Ditopic Ligands Bearing Phosphonic Acid or Phosphoester Units. Inorg. Chem..

[37] Verma P.K., Mahanty B., Bhattacharyya A., Matveev P.I., Borisova N.E., Kalmykov S.N., Mohapatra P.K. (2024). Pyridine Diphosphonate Ligand for Stabilization of Tetravalent Uranium and Neptunium in Aqueous Medium under Aerobic Conditions. Inorg. Chem..

[38] Bhattacharyya A., Ansari S.A., Matveev P.I., Zakirova G.G., Borisova N.E., Petrov V.G., Sumyanova T., Verma P.K., Kalmykov S.N., Mohapatra P.K. (2019). Unfolding the complexation and extraction of Am3+ and Eu3+ using N-heterocyclic aromatic diphosphonic acids: A combined experimental and DFT study. Dalton Trans..

[39] Charbonnière L.J., Weibel N., Retailleau P., Ziessel R. (2007). Relationship Between the Ligand Structure and the Luminescent Properties of Water-Soluble Lanthanide Complexes Containing Bis(bipyridine) Anionic Arms. Chem. Eur. J..

[40] Charpentier C., Salaam J., Lecointre A., Jeannin O., Nonat A., Charbonnière L.J. (2019). Phosphonated Podand Type Ligand for the Complexation of Lanthanide CationsPhosphonated Podand Type Ligand for the Complexation of Lanthanide Cations. Eur. J. Inorg. Chem..

[41] Purnama I., Salmahaminati, Abe M., Hada M., Kubo Y., Mulyana J.Y. (2019). Factors influencing the photoelectrochemical device performance sensitized by ruthenium polypyridyl dyes. Dalton Trans..

[42] Ruile S., Kohle O., Péchy P., Grätzel M. (1997). Novel sensitisers for photovoltaic cells. Structural variations of Ru(II) complexes containing 2,6-bis(1-methylbenzimidazol-2-yl)pyridine. Inorg. Chim. Acta.

[43] Wang L., Shaffer D.W., Manbeck G.F., Polyansky D.E., Concepcion J.J. (2020). High-Redox-Potential Chromophores for Visible-Light-Driven Water Oxidation at Low pH. ACS Catal..

[44] Zabri H., Gillaizeau I., Bignozzi C.A., Caramori S., Charlot M.-F., Cano-Boquera J., Odobel F. (2003). Synthesis and Comprehensive Characterizations of New cis-RuL2X2 (X = Cl, CN, and NCS) Sensitizers for Nanocrystalline TiO2 Solar Cell Using Bis-Phosphonated Bipyridine Ligands (L). Inorg. Chem..

[45] Zabri H., Odobel F., Altobello S., Caramori S., Bignozzi C.A. (2004). Efficient osmium sensitizers containing 2,2′-bipyridine-4,4′-bisphosphonic acid ligand. J. Photochem. Photobiol. A Chem..

[46] Rosser T.E., Windle C.D., Reisner E. (2016). Electrocatalytic and Solar-Driven CO2 Reduction to CO with a Molecular Manganese Catalyst Immobilized on Mesoporous TiO_2_. Angew. Chem. Int. Ed..

[47] Yao X., Zhang Q., Ho P.-Y., Yiu S.-C., Suramitr S., Hannongbua S., Ho C.-L. (2023). Development of Aldehyde Functionalized Iridium(III) Complexes Photosensitizers with Strong Visible-Light Absorption for Photocatalytic Hydrogen Generation from Water. Inorganics.

[48] Kobayashi A., Yamamoto N., Shigeta Y., Yoshida M., Kato M. (2018). Two-way vapochromism of a luminescent platinum(ii) complex with phosphonic-acid-functionalized bipyridine ligand. Dalton Trans..

[49] Montalti M., Wadhwa S., Kim W.Y., Kipp R.A., Schmehl R.H. (2000). Luminescent Ruthenium(II) Bipyridyl−Phosphonic Acid Complexes:  pH Dependent Photophysical Behavior and Quenching with Divalent Metal Ions. Inorg. Chem..

[50] Comby S., Imbert D., Chauvin A.-S., Bünzli J.-C.G., Charbonnière L.J., Ziessel R.F. (2004). Influence of Anionic Functions on the Coordination and Photophysical Properties of Lanthanide(III) Complexes with Tridentate Bipyridines. Inorg. Chem..

[51] Herath H.N.K., MacRae A.L., Ugrinov A., Morello G.R., Parent A.R. (2023). Electrochemical Properties of Ru Polypyridyl Phosphonates. Eur. J. Inorg. Chem..

[52] Ferrer Í., Fontrodona X., Roig A., Rodríguez M., Romero I. (2017). A Recoverable Ruthenium Aqua Complex Supported on Silica Particles: An Efficient Epoxidation Catalyst. Chem. Eur. J..

[53] Bonhôte P., Moser J.-E., Humphry-Baker R., Vlachopoulos N., Zakeeruddin S.M., Walder L., Grätzel M. (1999). Long-Lived Photoinduced Charge Separation and Redox-Type Photochromism on Mesoporous Oxide Films Sensitized by Molecular Dyads. J. Am. Chem. Soc..

[54] Amthor S., Keil P., Nauroozi D., Perleth D., Rau S. (2021). A Phosphonate Substituent in a 1,10-Phenanthroline Ligand Boosts Light-Driven Catalytic Water Oxidation Performance Sensitized by Ruthenium Chromophores. Eur. J. Inorg. Chem..

[55] Wang L., Xie Z.-L., Phelan B.T., Lynch V.M., Chen L.X., Mulfort K.L. (2023). Changing Directions: Influence of Ligand Electronics on the Directionality and Kinetics of Photoinduced Charge Transfer in Cu(I)Diimine Complexes. Inorg. Chem..

[56] Ma Y.-S., Tang X.-Y., Yin W.-Y., Wu B., Xue F.-F., Yuan R.-X., Roy S. (2012). Zinc and cadmium 2-pyrazinephosphonates: Syntheses, structures and luminescent properties. Dalton Trans..

[57] Ansari S.A., Mohapatra P.K., Sengupta A., Nikishkin N.I., Huskens J., Verboom W. (2014). An Insight into the Complexation of Pyrazine-Functionalized Calix [4]arenes with Am3+ and Eu3+—Solvent Extraction and Luminescence Studies in Room-Temperature Ionic Liquids. Eur. J. Inorg. Chem..

[58] Ansari S.A., Mohapatra P.K., Verboom W., Zhang Z., Dau P.D., Gibson J.K., Rao L. (2015). Binding of pyrazine-functionalized calix [4]arene ligands with lanthanides in an ionic liquid: Thermodynamics and coordination modes. Dalton Trans..

[59] Makwana M.V., dos Santos Souza C., Pickup B.T., Thompson M.J., Lomada S.K., Feng Y., Wieland T., Jackson R.F.W., Muimo R. (2023). Chemical Tools for Studying Phosphohistidine: Generation of Selective τ-Phosphohistidine and π-Phosphohistidine Antibodies. ChemBioChem.

[60] Bruyneel F., D’Auria L., Payen O., Courtoy P.J., Marchand-Brynaert J. (2010). Live-Cell Imaging with Water-Soluble Aminophenoxazinone Dyes Synthesised through Laccase Biocatalysis. ChemBioChem.

[61] Chen B., Ding J., Wang L., Jing X., Wang F. (2012). A solution-processable phosphonate functionalized deep-blue fluorescent emitter for efficient single-layer small molecule organic light-emitting diodes. Chem. Commun..

[62] Li X., Bai K., Wang S., Ding J., Wang L. (2019). An arylphosphine oxide and phosphonate combination as a solution processable electron injection layer for power-efficient PLEDs. J. Mater. Chem. C.

[63] Chen B., Zhao L., Ding J., Wang L., Jing X., Wang F. (2016). An alcohol-soluble and ion-free electron transporting material functionalized with phosphonate groups for solution-processed multilayer PLEDs. Chem. Commun..

[64] Li X., Yang L., Wang C., Wang S., Ding J. (2024). Bulky Passivation by an Electroactive Phosphonate Dendrimer for High-Performance Perovskite Quantum Dot Light-Emitting Diodes. J. Phys. Chem. C.

